# Neuroproteomic Analysis after SARS-CoV-2 Infection Reveals Overrepresented Neurodegeneration Pathways and Disrupted Metabolic Pathways

**DOI:** 10.3390/biom13111597

**Published:** 2023-10-30

**Authors:** Indranil Basak, Rhodri Harfoot, Jennifer E. Palmer, Abhishek Kumar, Miguel E. Quiñones-Mateu, Lucia Schweitzer, Stephanie M. Hughes

**Affiliations:** 1 Brain Health Research Centre, Department of Biochemistry, University of Otago, Dunedin 9016, New Zealand; 2 Department of Microbiology and Immunology, University of Otago, Dunedin 9016, New Zealandmquinon4@uwo.ca (M.E.Q.-M.); 3 Centre for Protein Research, University of Otago, Dunedin 9016, New Zealand

**Keywords:** NeuroCOVID, COVID-19, SARS-CoV-2, iPSC-derived human neurons and astrocytes, proteomics, mass spectrometry, apoptosis, neurodegeneration, metabolism

## Abstract

Besides respiratory illness, SARS-CoV-2, the causative agent of COVID-19, leads to neurological symptoms. The molecular mechanisms leading to neuropathology after SARS-CoV-2 infection are sparsely explored. SARS-CoV-2 enters human cells via different receptors, including ACE-2, TMPRSS2, and TMEM106B. In this study, we used a human-induced pluripotent stem cell-derived neuronal model, which expresses ACE-2, TMPRSS2, TMEM106B, and other possible SARS-CoV-2 receptors, to evaluate its susceptibility to SARS-CoV-2 infection. The neurons were exposed to SARS-CoV-2, followed by RT-qPCR, immunocytochemistry, and proteomic analyses of the infected neurons. Our findings showed that SARS-CoV-2 infects neurons at a lower rate than other human cells; however, the virus could not replicate or produce infectious virions in this neuronal model. Despite the aborted SARS-CoV-2 replication, the infected neuronal nuclei showed irregular morphology compared to other human cells. Since cytokine storm is a significant effect of SARS-CoV-2 infection in COVID-19 patients, in addition to the direct neuronal infection, the neurons were treated with pre-conditioned media from SARS-CoV-2-infected lung cells, and the neuroproteomic changes were investigated. The limited SARS-CoV-2 infection in the neurons and the neurons treated with the pre-conditioned media showed changes in the neuroproteomic profile, particularly affecting mitochondrial proteins and apoptotic and metabolic pathways, which may lead to the development of neurological complications. The findings from our study uncover a possible mechanism behind SARS-CoV-2-mediated neuropathology that might contribute to the lingering effects of the virus on the human brain.

## 1. Introduction

Severe acute respiratory syndrome coronavirus 2 (SARS-CoV-2), as the name suggests, causes respiratory symptoms that lead to coronavirus disease 2019 (COVID-19) [1]. The disease was first identified in the Wuhan district in China [1] and, within months, became a global pandemic, with close to 680 million total cases and over 6.8 million total deaths so far (COVID-19 Dashboard by the CSSE at JHU, accessed on 11 September 2023). More prominently, complications after COVID-19 infection remain manifold. Observations at the onset of the outbreak primarily listed respiratory symptoms like fever, cough, respiratory distress, and pneumonia. Nonetheless, the list of complications has expanded and gone well beyond respiratory symptoms. In 2020, one of the initial complications was the emergence of neurological symptoms in COVID-19 patients (NeuroCOVID), including dizziness, disturbed consciousness, headache, loss of smell and taste, seizures, encephalitis, and increased risk of stroke [2,3,4,5,6,7,8,9]. From the first SARS virus infection in the early 2000s, a study investigating organ distribution of the related SARS-CoV illustrated the presence of viral particles in brain autopsy tissue and spinal cord fluid [10]. Fast forward almost 20 years, a transmission electron microscopy of brain sections obtained via post-mortem examination from a male patient with Parkinson’s disease, who contracted the SARS-CoV-2 virus, showed the presence of the viral particles in frontal lobe brain sections [11]. Moreover, a 2021 study showed microvascular injury in the brain and olfactory bulb after SARS-CoV-2 infection [12]. With the continuing onslaught of SARS-CoV-2, more detailed studies started evolving around the virus and its effects on the brain. A 2021 article by Harappan and Yoo summarised a list of neurological conditions associated with COVID-19 patients until 2020, which included gustatory and olfactory dysfunctions, myalgia, headache, altered mental status, confusion, delirium, dizziness, stroke, cerebral venous thrombosis, seizures, meningoencephalitis, Guillain–Barre syndrome, Miller–Fisher syndrome, acute myelitis, and posterior reversible encephalopathy syndrome [13]. The symptoms were observed in both adults and children [14], and cortical haemorrhage was reported in the foetal brain [15]. A more extensive study in 2021 assessed neurological and psychiatric co-morbidity in more than 200,000 patients and found that the risks were more significant in patients with a more severe form of infection [16].

The UK Biobank reported a change in brain volume of COVID-19 patients in 2022 [17], which was also reported in patients who recovered from COVID-19 [18,19] and was associated with cognitive dysfunction [20]. Although conflicting studies have suggested that the virus does not infiltrate the brain directly [15,21], the changes in brain structure post-SARS-CoV-2 infection suggest neurological symptoms associated with COVID-19. Furthermore, a more recent study from September 2022 analysed patient data one year after SARS-CoV-2 infection and found that in the post-acute phase of COVID-19, the patients showed an increased risk of stroke, cognition and memory disorders, peripheral nervous system disorders, migraine, seizure, movement disorders, mental health disorders, musculoskeletal disorders, sensory disorders, Guillain–Barre syndrome, encephalitis, or encephalopathy [22]. Assessments of neurological symptoms in COVID-19 patients after recovery [22,23,24] have revealed that follow-up neurological assessments are critical to understanding the long-term effects of the virus, thereby making the virus a significant contributor to global health challenges. Genetic and molecular analyses followed by gene ontology analyses have revealed that SARS-CoV-2 infection showed altered cellular pathways that overlap with brain diseases such as Alzheimer’s disease, multiple sclerosis, and brain ageing [25,26,27,28,29].

In addition to the symptomatic studies over the last two years highlighting what SARS-CoV-2 seems to be doing to the brain, there has been a plethora of studies investigating how the virus affects the brain, leading to neurological symptoms in COVID-19 (or NeuroCOVID) patients [25,29,30,31,32,33,34,35,36]. A key finding indicates that the cytokine storm post-SARS-CoV-2 infection can lead to immune cell infiltration into the olfactory region, which could be an entry point to the brain [37,38]. Initially, it was described that SARS-CoV-2 uses the receptor protein ACE-2 and a transmembrane protein called TMPRSS2 on the host cell surface to gain entry to the cell [39]. Later, other receptors, such as ASGR1, BSG, NRP1, and TMEM106B, were identified, which allow the binding of the virus and entry into the host cells, sometimes independent of ACE-2 [40,41]. After entry into the host cells, the virus takes control of the host translation machinery [42], starting a cascade of changes in cellular pathways and leading to cell death. Multiple omics analyses have been performed on different human cell types to ascertain the cellular changes after SARS-CoV-2 infection. However, to our knowledge, less than ten research studies have assessed proteomic changes directly in brain cells post-SARS-CoV-2 infection [32,36,43,44,45]. Most studies have focussed on investigating transcriptomic and proteomic changes in the human body fluids, and there is a paucity of information on what proteomic changes occur in human brain cells, particularly in neurons, when exposed to SARS-CoV-2. The information from the neuroproteomic analysis can reveal the dysregulated neuronal pathways that may explain the neurological symptoms in COVID-19 patients.

Ideally, investigating the disturbed cellular and molecular pathways in brain cells from living patients affected by COVID-19 would reveal the mechanisms behind the neuropathology and can lead to therapy. However, there is limited access to live human brain cells, but skin cell-derived induced pluripotent stem cells (iPSCs) offer an excellent platform to generate human brain cells on a dish to investigate the dysfunctional pathways after SARS-CoV-2 infection. Our transcriptomic data in our well-established iPSC-derived human cortical-like glutamatergic neuronal model [46,47] show the expression of ACE2 along with other receptors. These expression data were supported by the publicly available RNA-sequencing database (https://ineuronrnaseq.shinyapps.io/rnaseq_app/, accessed on the 20 April 2021) on these iPSC-derived cortical-like glutamatergic neurons (Figure S1) [48]. The presence of these entry points for SARS-CoV-2 seems to make neurons vulnerable to the virus. Therefore, we aimed to delineate the neuronal pathologies after exposure to SARS-CoV-2, and in this study, we present unique neuroproteomic signature altering key neuronal pathways that could explain the neurological complications associated with COVID-19.

## 2. Materials and Methods

### 2.1. Generation of Induced Pluripotent Stem Cell-Derived Human Neurons

**Figure 1:**
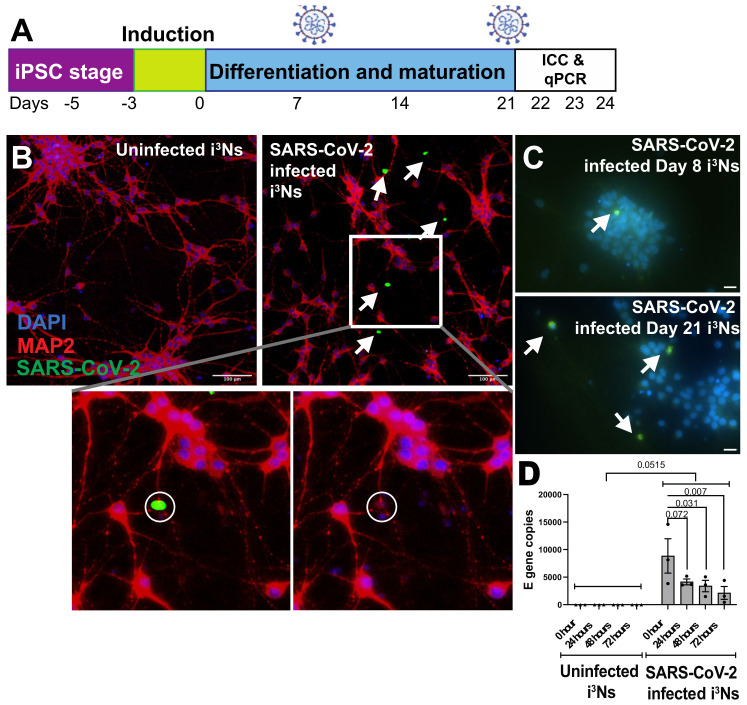
SARS-CoV-2 virus infects human iPSC-derived neurons. (**A**) iPSCs were differentiated into cortical-like glutamatergic neurons (i^3^Ns) and infected on Day 21, followed by immunocytochemistry analysis on Day 22. To assess infection in immature i^3^Ns, infection was carried out on Day 8 and immunocytochemistry on Day 9. Finally, for RT-qPCR analysis of viral replication, i^3^Ns were infected on Day 21, followed by analysis on Days 22, 23, and 24. (**B**) i^3^Ns showed infection 24 h post-infection, as shown by the white arrows. Inset shows that the infected nucleus (DAPI stain) looked fragmented compared to the uninfected nucleus. (**C**) Day 21 i^3^Ns showed more SARS-CoV-2 infected cells than Day 8 i^3^Ns. (**D**) RT-qPCR analysis of the E gene in infected i^3^Ns showed no virus replication in the i^3^Ns.

For this study, an established protocol to generate pure human cortical-like glutamatergic neurons from induced pluripotent stem cells (iPSCs) was used [46]. In short, using the doxycycline-mediated expression of the transcription factor *neurogenin-2*, the iPSCs were differentiated into mature isogenic, integrated, and inducible pure human cortical-like glutamatergic neurons (i^3^Ns). The i^3^Ns express mature neuronal markers after 2 weeks [46,47] and show electrophysiological activity [47,49]. An early, immature stage (Day 8) for i^3^Ns was chosen in addition to a late, mature stage (Day 21) of i^3^Ns to test whether the SARS-CoV-2 virus preferentially infects immature versus mature neurons. The i^3^Ns were exposed to SARS-CoV-2 at these two different time points (Days 8 and 21, Section 2.3) and two different multiplicities of infection (MOI) (Figure 1A). 24, 48, and 72 h post-exposure, the i^3^Ns were harvested for RNA isolation, and the RNA was used to quantify SARS-CoV-2 replication (Section 2.4). Three-week-old i^3^Ns were exposed to SARS-CoV-2 (Section 2.3) for either immunochemistry or proteomic experiments. To test the effect of hypoxia and SARS-CoV-2 together, the i^3^Ns were incubated with cobalt chloride (Sigma-Aldrich, Castle Hill, Australia; cat# 232696) at two different concentrations (100 μM and 200 μM, hypoxia confirmed by HIF1-α immunocytochemistry) for 24 h, followed by exposure to SARS-CoV-2 and immunocytochemistry as described above.

### 2.2. Generation of Induced Pluripotent Stem Cell-Derived Human Astrocytes

Following Canals et al. [50] and Fernandopulle et al. [47], the transcription factor *Nfib* was stably integrated into WTC11 iPSCs under a doxycycline-inducible promoter (Basak, Hughes et al., manuscript in preparation). The Nfib cassette, based on the plasmids (Addgene 64900 and 105840), consisted of mApple and puromycin selection markers. The iPSCs with stably integrated Nfib were sorted for mApple via fluorescence-activated cell sorting and selected for puromycin, yielding a pure iPSC population with integrated Nfib. The insertion of Nfib was also tested via PCR and sequencing, followed by differentiation of the Nfib-iPSCs into mature astrocytes (iAs) in 17 days (Figure S2A), following the original protocol [50]. The Day 17 iAs showed expression of S100β, known to be highly expressed in mature human astrocytes [51]. Like the neuronal infection, the iAs were exposed to SARS-CoV-2 (Section 2.3) at 10 MOI (Figure S2A). 24 h post-SARS-CoV-2 exposure, the iAs were used for immunochemistry to detect SARS-CoV2 infection.

### 2.3. SARS-CoV-2 Production and Infection of i^3^Ns and iAs 

SARS-CoV-2 isolate hCoV-19/New Zealand/NZ1_patient/2020 was produced in Vero E6/TMPRSS2 cells as described [52]. The viral stock was titrated by determining tissue culture dose for 50% infectivity (TCID_50_) in triplicate with cytopathic effect (CPE) as the end-point using the Reed and Muench method [53] and expressed as TCID_50_ per millilitre (TCID_50_/mL). The SARS-CoV-2 titre was used to determine the appropriate MOI to be added to the iPSC-derived i^3^Ns (Section 2.1) or iAs (Section 2.2). Younger (Day 8) and mature (Day 21) i^3^Ns were exposed to SARS-CoV-2 using two different MOIs, i.e., 2 and 10, for 24 h, followed by fixing the cells with 4% paraformaldehyde and immunocytochemistry (Figure 1B,C). Similarly, Day 17 iAs were exposed to SARS-CoV-2 at 10 MOI, followed by 24 h infection and immunocytochemistry (Figure S2A). 

### 2.4. Assessment of SARS-CoV-2 Infection and Replication

To test the ability of SARS-CoV-2 to infect i^3^Ns and/or iAs, cells were fixed 24 h post-exposure and used for immunocytochemistry as before [46]. Primary antibodies were used to detect SARS-CoV-2 (mouse anti-SARS-CoV-2 nucleoprotein antibody, Sapphire Biosciences, Redfern, Australia, cat# 35-579, dilution—1:500), neurons (guinea pig anti-MAP2 antibody, Synaptic Systems, Gottingen, Germany, cat# 188004, 1:2000) and astrocytes (rabbit anti-S100β antibody, Abcam, Burlingame, CA, USA, cat# ab52642, dilution—1:400). Secondary antibodies used were similar to [46], followed by counterstaining with 4′, 6-diamidino-2-phenylindole (DAPI) (Sigma-Aldrich, Castle Hill, Australia; cat# D9542, dilution—1:10,000 from 1 mg/mL stock solution), as previously described [46]. After immunocytochemistry, the i^3^Ns and iAs were imaged on a Nikon Ti2 Inverted microscope (Nikon, Tochigi, Japan).

To test whether SARS-CoV-2 can replicate within post-mitotic i^3^Ns, three-week-old i^3^Ns were exposed to SARS-CoV-2 (Section 2.3) and incubated with the virus for 24, 48, and 72 h. Post-viral exposure, the i^3^Ns were washed with PBS (ThermoFisher Scientific, Auckland, New Zealand, cat#70011044) to rid them of the free-floating SARS-CoV-2 or non-internalised SARS-CoV-2 and the E gene quantification represented true neuronal infection. Then, the i^3^Ns were harvested with Accutase (ThermoFisher Scientific, Auckland, New Zealand, cat# A1110501) and total RNA was isolated using the TRIzol RNA isolation method [54] and PureLink RNA isolation kit (ThermoFisher Scientific, Auckland, New Zealand, cat# 12183018A), following manufacturer’s instructions. The RNA was quantified using a NanoDrop One spectrophotometer (ThermoFisher Scientific, Madison, WI, USA) and treated with DNAseI (ThermoFisher Scientific, Auckland, New Zealand, cat# 18-068-015) following our previously established protocol [55]. RT-qPCR was performed using a LightCycler 480 Instrument (Roche, Auckland, New Zealand), qScript XLT 1-Step RT-qPCR ToughMix Low ROX (Quantabio, Beverly, CA, USA, cat# 84385), and primers designed to detect the E gene (envelope gene) of SARS-CoV-2 (primer sequences as described [56]. The expression of the E gene was normalised to GAPDH (primer sequences: forward primer—5′ CCACTCCTCCACCTTTGAC 3′, reverse primer—5′ ACCCTGTT GCTGTAGCCA 3′), and analysis was performed using the Pfaffl method [57].

### 2.5. Neuroproteomic Analysis of SARS-CoV-2 Infected Human Neurons

The unexposed i^3^Ns and i^3^Ns exposed to SARS-CoV-2 (at 10 MOI) were used for the first set of neuroproteomic analyses (Figure S3). Because of the known effect of cytokines on neurons from the literature, human lung epithelial Calu-3 cells were infected with SARS-CoV-2 (10 MOI), and the i^3^Ns were treated either with pre-conditioned media from uninfected or infected Calu-3 cells (Figure S3). The SARS-CoV-2 exposure to i^3^Ns and treatment of the i^3^Ns with the pre-conditioned Calu-3 media was carried out for 24 h. Following SARS-CoV-2 exposure (both direct and with pre-conditioned media), all i^3^Ns from four conditions (Figure S3) were used for mass spectrometric analysis. The lists of differentially expressed proteins were used for (i) identifying protein–protein interaction using StringDB [58] and (ii) gene ontology analysis using Metascape [59], followed by verification on Enrichr [60] and DAVID [61] and visualisation using Cytoscape [62]. For STRING analysis, the upregulated or downregulated proteins were compiled into two different lists (from Tables S1 and S2), and these lists were used to query the protein–protein interactions on Cytoscape. For Cytoscape visualisation, a full STRING network was investigated with a confidence (score) cutoff of 0.4 and a maximum additional interactors cutoff of 10 from the 1st shell. For gene ontology analyses, the upregulated and downregulated protein lists were used to ascertain the changing molecular functions and biological pathways with a cutoff of *p* < 0.05.

### 2.6. Label-Free Proteomic Analysis of SARS-CoV-2 Infected Neurons

The neuron samples were lysed in an SDS containing lysis buffer (5% SDS, 50 mM TEAB). Genomic DNA was degraded with a nuclease, Denarase (c-LEcta, Leipzig, Germany). A BCA protein estimation assay (ThermoFisher Scientific, Auckland, New Zealand, cat# 23225) was used to normalise the protein amount to 100 µg in all samples. Further, reduction and alkylation were carried out using 5 mM Tris(2-carboxyethyl)phosphine hydrochloride (TCEP) (Sigma-Aldrich, Castle Hill, Australia; cat# C4706) and 10 mM iodoacetamide (Sigma-Aldrich, Castle Hill, Australia; cat# GERPN6302), respectively. Samples were then processed using the S-trap micro spin trap column (ProtiFi, Fairport, NY, USA) according to the manufacturer’s protocol. The proteins on the column were tryptically digested, and cleaved peptides were eluted from the column for the proteomics analysis (protifi.com/pages/protocols, accessed on 11 September 2023). 

The peptides were chromatographically separated on a 20 cm emitter-tip column (75 μm ID fused silica tubing (CoAnn Technologies, Richland, WA, USA) in-house packed with 3 µM C-18 Luna material (Phenomenex, Torrance, CA, USA) using an Ultimate 3000 uHPLC system (ThermoFisher Scientific, Waltham, MA, USA). The peptides were eluted from the column using a two-hour method with a reverse phase acetonitrile (ACN) gradient. The gradient consisted of the following steps: 5% to 25% ACN in 84 min, 25% to 40% in 10 min, and 40% to 99% ACN in 5 min. Peptides were measured by an LTQ Orbitrap XL (ThermoFisher Scientific, Waltham, MA, USA) mass spectrometer at a resolution of 60,000 @ *m*/*z* 400. The 10 strongest precursor ions between 400–2000 *m*/*z* were selected for collision-induced dissociation (CID) fragmentation in the ion trap. A normalised collision energy was set at 35% with an AGC target of 2 × 10^5^. Dynamic exclusion was enabled with 2 repeat counts during 90 s and an exclusion period of 120 s. MS raw data were analysed with the Proteome Discoverer software (version: 2.5, ThermoFisher Scientific, Waltham, MA, USA). Spectra were searched against the human proteome (Uniprot.org) sequence database using the Sequest search engine node. The search was set up to look for the semi-tryptic peptides. In further search settings, carbamidomethyl cysteine was included as static modification, and deamidation of asparagines and glutamines were included as variable modifications. The precursor mass tolerance and the maximum fragment mass error threshold were set at 10 ppm and 0.6 Da, respectively. The false discovery rate (FDR) threshold was set at 1% within the percolator node. The resulting quantitative data was normalised on the sum of abundances from all peptides detected from all samples. The relative abundance of the proteins was calculated with the top 3 approaches [63], where the average abundance of the three most abundant peptides for a particular protein was used. The resulting abundance values were used to calculate the protein abundance ratio between infected vs. non-infected neurons to obtain the list of regulated proteins. The data were exported to Excel for further statistical analysis.

### 2.7. Statistical Analysis

All experiments were performed in experimental triplicate and analysed on GraphPad Prism (GraphPad, San Diego, CA, USA). For RT-qPCR analysis to test viral replication, two-way ANOVA was used to assess statistical significance between days post-infection and the measured number of E gene copies. To measure the increase in E gene copies in infected i^3^Ns versus uninfected i^3^Ns, a paired student’s *t*-test was used. For the proteomic analysis, a two-tailed student’s *t*-test was used to identify statistically significant differentially expressed up- and downregulated proteins. For all the analyses and determining significantly upregulated and downregulated pathways, biological processes, and molecular functions from the proteomic analysis, *p*-value < 0.05 was considered statistically significant. Data are presented as mean ± standard error of mean (SEM). * *p* < 0.05, ** *p* < 0.01, *** *p* < 0.001.

## 3. Results

### 3.1. SARS-CoV-2 Infects iPSC-Derived Human Neurons and Astrocytes

To test whether SARS-CoV-2 is able to infect iPSC-derived human neuronal cells, mature i^3^Ns were exposed to SARS-CoV-2 at two different MOIs. Cells exposed to an MOI of 2 failed to show signs of SARS-CoV-2 infection; however, using a higher MOI of 10, infection in i^3^Ns was detected (Figure 1B), albeit low compared to other cells highly susceptible to SARS-CoV-2 infection, such as Vero and VeroE6/TMPRSS2 [52]. Next, to test whether susceptibility to SARS-CoV-2 changes between immature (Day 8) and mature (Day 21) i^3^Ns, 10 MOI SARS-CoV-2 was added to immature and mature i^3^Ns. The mature neurons showed higher infectivity than the immature neurons (Figure 1C). To evaluate the role of hypoxia in neuron susceptibility to SARS-CoV-2 infection, the i^3^Ns were incubated with cobalt chloride to induce hypoxia, which was confirmed via HIF1-α staining, followed by exposing the i^3^Ns to SARS-CoV-2 to 10 MOI. The hypoxia treatment did not increase the ability of SARS-CoV-2 to infect i^3^Ns (data not shown). 

As an alternative model to test SARS-CoV-2 infection, human iPSC-derived astrocytes (iAs) were exposed to SARS-CoV-2 at 10 MOI. Similar to the i^3^Ns, the iAs also showed low infection via SARS-CoV-2, contrary to what has been previously described [31]. However, unlike neuronal nuclei, astrocyte nuclei looked intact (Figure S2B). This phenotype suggests that although SARS-CoV-2 infects both neurons and astrocytes, perhaps neurons are more vulnerable to apoptosis upon SARS-CoV-2 infection than the astrocytes. 

### 3.2. SARS-CoV-2 Does Not Replicate within Human Neurons

SARS-CoV-2 infects and replicates in different human cell types by hijacking the cellular machinery [42,64]. The amplification of the E gene, which encodes for the envelope protein of SARS-CoV-2, was tested using RT-qPCR at different time points post-infection to determine whether SARS-CoV-2 can replicate in the i^3^Ns. Our RT-qPCR results showed near significant (*p*-value = 0.0515) increased expression of the E gene in infected i^3^Ns versus the uninfected i^3^Ns (Figure 1D). We also noticed a significant decrease in E gene copies between 48 and 72 h post-infection versus 0 h post-infection (*p*-value 0.031 and 0.007, respectively) in i^3^Ns, most likely indicating initial SARS-CoV-2 infection with no viral production (Figure 1D). These data suggest that the neurons do not support SARS-CoV-2 replication, demonstrating the low infection rate observed in our neuronal model (Figure 1B,C). Perhaps the post-mitotic nature of the i^3^Ns could make them resistant to viral replication. A similar observation was made by Ramani et al. [65] when they exposed human brain organoids to SARS-CoV-2. 

### 3.3. SARS-CoV-2 Infection in Neurons Shows Distinct Changes in Neuronal Proteome

A mass spectrometric analysis of i^3^Ns exposed to SARS-CoV-2 compared to uninfected i^3^Ns was performed. With neuroinflammation being a major symptom in neurological cases of COVID-19 and our results showing low SARS-CoV-2 infection in the neuronal model, a second set of mass spectrometric analyses comparing SARS-CoV-2-infected vs. uninfected i^3^Ns was performed, where the i^3^Ns were treated with pre-conditioned media from infected and uninfected human lung epithelial Calu-3 cells (Figure S3). Calu-3 cells are much more readily infected by SARS-CoV-2 than neurons [66]. The pre-conditioned media from infected Calu-3 cells presumably contains inflammatory molecules that could potentially impact neuronal health and cause molecular changes leading to neuropathology. Therefore, for the second part of the proteomic analysis, i^3^Ns were treated with pre-conditioned media from infected Calu-3 cells compared to i^3^Ns treated with media from uninfected Calu-3 cells (Figure S3).

**Figure 2:**
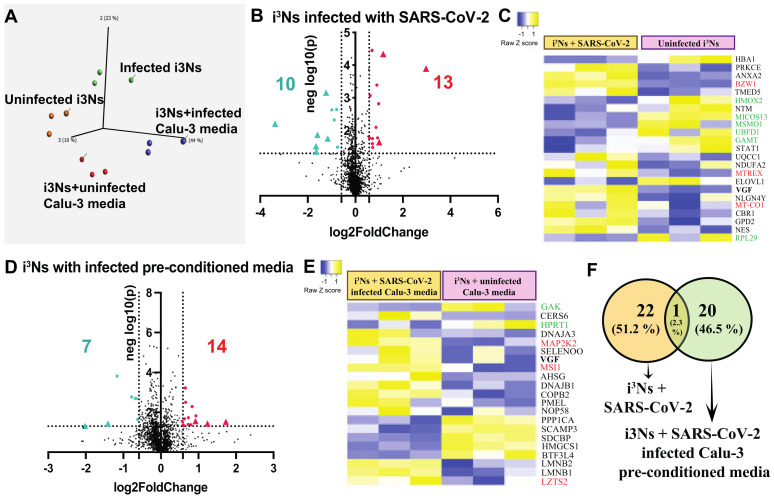
Proteomic analysis of SARS-CoV-2 infected human iPSC-derived neurons. (**A**) PCA plot shows the infected i^3^Ns segregated and clustered away from the uninfected i^3^Ns. (**B**) Mass spectrometric analysis of i^3^Ns infected directly with SARS-CoV-2 showed 13 upregulated and 10 downregulated proteins compared to uninfected i^3^Ns. (**C**) Heatmap showing differentially expressed proteins in infected (3 left columns) versus uninfected (3 right columns) i^3^Ns. Each column represents experimental replicates. Green text indicates downregulated proteins with > 2-fold change, while red text indicates upregulated proteins with > 2-fold change. (**D**) Mass spectrometric analysis of i^3^Ns treated with pre-conditioned SARS-CoV-2 infected Calu-3 media showed 14 upregulated and 7 downregulated proteins compared to i^3^Ns treated with uninfected Calu-3 media. (**E**) Heatmap showing differentially expressed proteins in i^3^Ns with infected (3 left columns) versus uninfected Calu-3 media (3 right columns). Each column represents experimental replicates. Green text indicates downregulated proteins with > 2-fold change, while red text indicates upregulated proteins with > 2-fold change. (**F**) Overlap of the two datasets (i^3^Ns directly infected versus i^3^Ns treated with pre-conditioned media compared to their respective controls) shows 1 protein (VGF) upregulated in both datasets.

The i^3^N lysates from the four groups, i.e., (i) uninfected i^3^Ns, (ii) i^3^Ns infected directly with SARS-CoV-2 at 10 MOI, (iii) i^3^Ns treated with media from uninfected Calu-3 cells, and (iv) i^3^Ns treated with pre-conditioned media from SARS-CoV-2 infected Calu-3 cells were subjected to mass spectrometric analysis. Using mass spectrometry, we identified more than 1700 proteins from all the conditions mentioned above, and each group clustered and segregated from each other on a PCA plot (Figure 2A), particularly the uninfected versus infected i^3^Ns. With a fold change cutoff of ± 1.5 and a *p*-value cutoff of <0.05, 13 upregulated and 10 downregulated proteins were identified as differentially expressed in i^3^Ns infected with SARS-CoV-2 compared to uninfected i^3^Ns (Figure 2B,C, Table S1). With more stringent criteria, i.e., a fold change cut-off of 2, only three upregulated and six downregulated proteins were found to be differentially expressed in i^3^Ns infected with the virus compared to uninfected i^3^Ns (Figure S4A). Applying the same initial cutoff, 14 upregulated and 7 downregulated proteins were identified to be differentially expressed in i^3^Ns with pre-conditioned infected Calu-3 media compared to i^3^Ns with uninfected Calu-3 media (Figure 2D,E, Table S2). More stringent criteria of fold change > 2 showed that only three upregulated and two downregulated proteins were found to be differentially expressed in i^3^Ns with pre-conditioned infected Calu-3 media compared to i^3^Ns with uninfected Calu-3 media (Figure S4B). Interestingly, only one protein (VGF nerve growth factor) overlapped between the two datasets (Figure 2F). 

### 3.4. SARS-CoV-2 Infection Affects Apoptotic and Metabolic Pathways in Neurons

**Figure 3:**
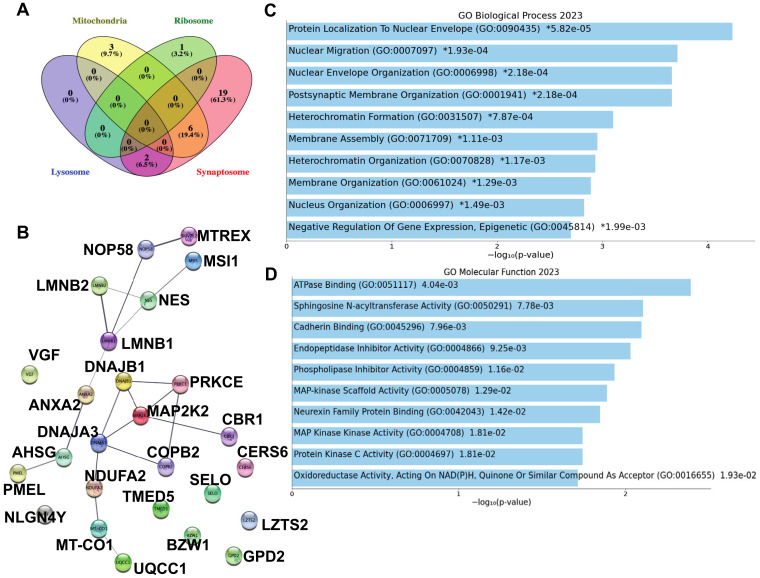
Analysis of upregulated proteins in infected i^3^Ns. (**A**) Combining all the up and downregulated proteins in SARS-CoV-2 infected i^3^Ns (direct and treated with pre-conditioned media), 29 proteins were found to be associated with the synaptosome, while 9 mitochondrial and 2 lysosomal proteins were observed to be altered. (**B**) Protein–protein interaction of all upregulated proteins in infected i^3^Ns showed some interactions related to apoptosis, neurodegeneration pathways, and chemical carcinogenesis—reactive oxygen species. (**C**) Changes in biological processes for upregulated proteins in infected i^3^Ns. * indicates the top statistically significant processes. (**D**) Changes in molecular functions for upregulated proteins in infected i^3^Ns.

As the number of differentially expressed proteins was smaller than in other proteomic studies, all the upregulated (26 in total) and all the downregulated (17 in total) proteins were combined in two lists. Upon further investigation of the localisation of the two lists of differentially expressed proteins, 27 proteins were found to be associated with the synaptosome, while 9 mitochondrial and 2 lysosomal proteins showed changes in the i^3^Ns either infected with SARS-CoV-2 directly or treated with the pre-conditioned infected Calu-3 media (Figure 3A). Association with the synaptosome indicates that neuronal communication might have been affected post-SARS-CoV-2 infection, while changes in mitochondrial and lysosomal proteins indicate an alteration in neuronal energy homeostasis, metabolism, and waste clearance activities in the neurons.

The upregulated proteins showed clustering and interactions (Figure 3B); however, gene ontology analysis did not reveal many significant pathways to be differentially regulated. Among the upregulated pathways (from Metascape [59]), chemical carcinogenesis—reactive oxygen species, apoptosis, neurodegeneration pathways, and membrane organisation, particularly mitochondrion organisation) (Table S3) are noteworthy. After running the same analysis with all the upregulated proteins on DAVID and Enrichr, the pathways mentioned above were confirmed, in addition to the intellectual disability pathway being enriched with the proteins upregulated in infected i^3^Ns. An extended STRING analysis by including 10 interactors from the first shell (differentially expressed upregulated proteins) revealed a more extensive protein–protein interaction network (Figure S5A). Most of the proteins in the network (Figure S5A) seemed to be involved in apoptosis (Figure S5B), reconfirming our observation of the upregulated apoptosis-related proteins in neurons infected with the SARS-CoV-2 virus. The upregulated protein VGF from directly and indirectly infected i^3^Ns was found to be involved in synaptic transmission (Table S3). Assessments of the biological processes changing with the upregulated proteins in SARS-CoV-2-infected i^3^Ns revealed protein localisation and membrane organisation to be affected (Figure 3C, Table S4). Finally, ATPase binding and kinase activity were upregulated molecular functions among other functions in infected i^3^Ns (Figure 3D, Table S5).

**Figure 4:**
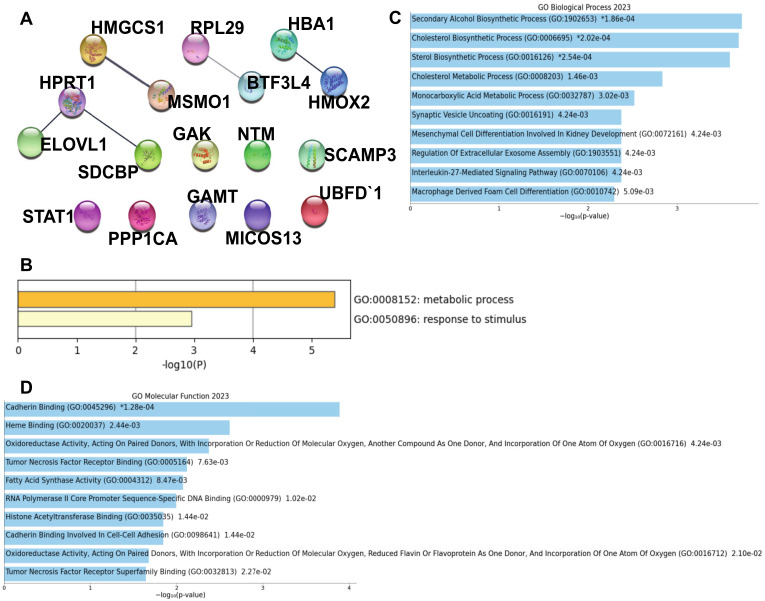
Analysis of downregulated proteins in infected i^3^Ns. (**A**) Protein–protein interaction of all downregulated proteins in infected i^3^Ns showed nominal interactions. (**B**) Metabolic processes were the most significant pathway to show change associated with downregulated proteins. (**C**) Changes in biological processes for downregulated proteins in infected i^3^Ns. * indicates the top statistically significant processes. (**D**) Changes in molecular functions for downregulated proteins in infected i^3^Ns.

The downregulated proteins showed almost no clustering and fewer interactions (Figure 4A) than the upregulated protein group. However, gene ontology analysis revealed metabolic pathways to be a significantly downregulated pathway (from Metascape) in infected i^3^Ns (Figure 4B, Table S6). DAVID and Enrichr analyses confirmed metabolism, particularly lipid metabolism, as the most significantly downregulated pathway in the infected i^3^Ns. Extended STRING analysis to include 10 interactors from the 1st shell (differentially expressed upregulated proteins) showed better clustering (Figure S6A) compared to what was observed in Figure 4A. Furthermore, STRING analysis with the extended interactors followed by Cytoscape visualisation also revealed clusters of downregulated proteins in the infected neurons that are involved in metabolism (Figure S6B), axon guidance (Figure S6C), cholesterol biosynthesis (Figure S6D) and cell response to stress (Figure S6E). Assessments of the biological processes changing with the downregulated proteins in infected i^3^Ns revealed cholesterol biosynthesis to be affected (Figure 4C, Table S7). Finally, Cadherin binding was one of the downregulated molecular functions in infected i^3^Ns (Figure 4D, Table S8).

## 4. Discussion

The main goal of our study was to understand the pathological mechanisms underlying the adverse neurological symptoms after SARS-CoV-2 infection. To achieve this, we used a model based on iPSC-derived human cortical-like glutamatergic neurons, which were exposed to the COVID-19-causing SARS-CoV-2 virus prior to assessing potential neuronal damage. Molecular and immunocytochemistry experiments showed that our neuron model had limited susceptibility to SARS-CoV-2 infection, with no virus production compared to multiple other cell types, including human respiratory epithelial cells [66]. Interestingly, the neurons that got infected with SARS-CoV-2 did not survive, evident from the fragmented DAPI-stained DNA (Figure 1B, second inset) and supported by the upregulation of apoptosis-related proteins from the proteomic analysis of infected neurons (Table S3). One caveat to this SARS-CoV-2 infection of i^3^N experiment was that the number of neurons being infected by the virus could not be quantified, as the neuronal nucleus (DAPI staining) looked fragmented after the viral infection (Figure 1B, second inset). A 2021 paper [65] also observed a low infection rate and similar disintegrated nucleus phenotype in brain organoid neurons when infected with 10 MOI SARS-CoV-2. Another 2023 paper [34] also reported a similar low infection rate in neurons. However, a study in iPSC-derived sensory neurons showed a higher SARS-CoV-2 infection rate at 1 MOI [67], possibly because of the differences in the model, neuronal culture conditions, and the molecular and cellular architecture of sensory neurons versus cortical neurons. An increase in apoptotic pathways has also been described in other studies investigating changes in plasma proteins from COVID-19 patients [33,43].

SARS-CoV-2 did not replicate in our neuronal model, also shown by [65,67], perhaps due to the post-mitotic nature or other evading mechanisms in the neurons. With systemic hypoxia associated with COVID-19 disease, a study showed that brain hypoxia has been observed in a smaller set of COVID-19 patients [68]. The neurons may be evading the viral infection, and the presence of hypoxia does not make the neurons vulnerable, suggesting that there are other molecular mechanisms responsible for neuropathology after SARS-CoV-2 infection. Similarly, iPSC-derived astrocytes showed low SARS-CoV-2 infection compared to other cells like VeroE6 or VeroE6/TMPRSS2 [52]. Previous research involving glial cells and SARS-CoV-2 has shown differential infection and contradictory results. A study involving 52 COVID-19 patients from the First Polish Brain Bank showed imminent effects on astroglial proliferation [69]. Two iPSC-derived organoid studies revealed that SARS-CoV-2 uses the Neuropilin-1 receptor to infect astrocytes at a rate more than we have observed, and SARS-CoV-2 infection enhances astrocyte reactivity [70,71]. Both studies used iPSC-derived organoids hosting other brain cells compared to our iPSC-derived model representing a pure culture of human astrocytes, which may be the reason behind the different infection rates observed in our study. However, two other studies showed that SARS-CoV-2 hardly infects iPSC-derived astrocytes [72] and does not replicate over time in primary human brain-extracted astrocytes [73]. These studies, along with our observation in iPSC-derived astrocytes, indicate a contradicting astrocyte infection pattern of SARS-CoV-2. However, there is a unanimous consensus that human neurons are barely infected by SARS-CoV-2 [65,67,72], as observed in our study. Another type of brain cells, microglia, are readily infected by SARS-CoV-2, as shown in multiple studies, which could lead to inflammatory responses detrimental to the neurons [74,75,76,77]. Mild respiratory COVID-19 can cause microglial activation-mediated neuroinflammation and impair neurogenesis [76]. Furthermore, COVID-19 patients with no neurological symptoms can also show neuronal and glial degeneration [74]. Another study used immunohistochemical analyses of post-mortem brain slices from COVID-19 patients to show that SARS-CoV-2 infection was associated with active ramified microglia, which may influence neurogenesis [78]. A more in-depth analysis of neurogenesis and microglial activation after SARS-CoV-2 infection revealed that an elevated level of a particular cytokine, CCL11, enhances microglial reactivity and impairs neurogenesis [76]. This differential infection of brain cells via SARS-CoV-2 brings us back to the neurotropism question of SARS-CoV-2, which is still underexplored. Perhaps the different cellular and molecular architecture is responsible for making the brain cells differentially vulnerable to the SARS-CoV-2 virus, and therefore, determines which cells are more affected in COVID-19 patients.

With thousands of studies dissecting the molecular mechanisms leading to neurological complications after COVID-19, it is surprising how few studies have investigated proteomic changes in brain cells. The majority of the proteomic studies involved in NeuroCOVID research have been conducted in serum [79], plasma [33,80], or cerebrospinal fluid (CSF) [35,36,81] from COVID-19 patients. One of these studies [36] showed decreased VGF (VGF nerve growth factor) expression in CSF from COVID-19 patients. This observation contradicts our finding, i.e., the upregulation of VGF in neurons exposed directly or indirectly to SARS-CoV-2 (Figure 2C,E,F). Albeit Reinhold et al. [36] used CSF, while our study was performed on human neurons, a GEO dataset (GSE37827) also revealed the identification of VGF mRNA alteration after SARS-CoV infection in Calu-3 cells. VGF is known to be upregulated in HIV-associated neuropathy [82] and can lead to weakness. In our case, another RNA virus (SARS-CoV-2) seems to increase VGF, which could contribute to weakness and fatigue symptoms observed in COVID-19 patients [14,24,31,68,83]. Furthermore, VGF is associated with synaptic transmission (Table S3), and a study in two different brain regions from COVID-19 patients showed enriched synaptic neurotransmitter release [44].

Similarly, another NeuroCOVID proteomic study on patient CSF showed AHSG (a glycoprotein) to be downregulated in COVID-19 patients, contrary to our upregulation of AHSG in the infected neurons (Figure 2E). AHSG is required for brain development and is associated with alopecia and mental retardation syndrome [84] and Alzheimer’s disease [85]. As discussed earlier, SARS-CoV-2 infection shares cellular mechanisms with Alzheimer’s disease [29]. It is possible that an increase in AHSG post-infection in neurons may predispose the neurons to apoptotic pathways, as seen in neurodegenerative diseases. A 2021 study [86] showed that COVID-19 patients were associated with impaired amyloid processing measured in CSF and serum, and this phenomenon might contribute to neurological symptoms post-SARS-CoV-2 infection. Additional studies have also explored a possible link between COVID-19 and Alzheimer’s disease [25,29]. Similar to these studies, our proteomic dataset revealed upregulated proteins associated with neurodegenerative pathways, including Alzheimer’s disease (Table S3). The increased expression of *MT-CO1* (mitochondrial COX1) has been reported in blood samples from Alzheimer’s disease [87]. Another mitochondrial protein, DNAJA3 (also known as TID1 or HSP40), is upregulated in the infected neurons, and an increase in DNAJA3 expression is not only associated with increased neuronal apoptosis but also has been shown to be increased in Alzheimer’s disease [88]. MAP2K2 (MAP kinase) upregulation is associated with the hyperphosphorylation of tau, contributing to the development of Alzheimer’s disease [89]. The reduced expression of PPP1CA also contributes to tau hyperphosphorylation, and Alzheimer’s disease brain samples show reduced PPP1CA expression [90]. Therefore, our observation of the increased expression of MT-CO1 and MAP2K2 and decreased expression of PPP1CA (Figure 2C,E) in infected neurons seems to support the theory that SARS-CoV-2 infected neurons may be predisposed to neurodegeneration.

A recent study involving proteomics of human tissue showed the effect of inflammation post-infection on the basal ganglia and the brain stem [44] and suggested changes in trafficking in AMPA receptors via inflammation, along with increased abundance of protein kinases PRKCG, PRKCB, and CAMK2A/B. Protein kinases regulate AMPA receptor signalling and trafficking [91]. Although our analysis did not identify AMPA receptors, a protein kinase PRKCE was upregulated in infected neurons (Figure 2C), and PRKCE is involved in the regulation of trans-synaptic signalling, particularly AMPA receptor signalling [92]. PRKCE was also upregulated in nasopharyngeal swabs from COVID-19 patients [93]. SARS-CoV-2 is known to hijack the cellular kinase system to facilitate viral RNA synthesis [94,95], and our study showed increased PRKCE expression (Figure 2C) and kinase activity to be an overrepresented molecular function in the infected neurons (Figure 3D), suggesting that the neuronal kinase activity was affected either via direct infection or via indirect infection. In our analysis, one of the overrepresented pathways was mitochondrion organisation (Table S3), whereas Schweizer et al. showed increased mitochondrial protein translation [44]. Although both pathways consist of distinct proteins, it is known that SARS-CoV-2 can hijack the host cell mitochondria to viral advantage [96] as well as induce host mitochondrial dysfunction [97]. In fact, in our analysis, eight (out of nine) mitochondrial proteins identified were upregulated in the SARS-CoV-2 infected neurons (Figure 3A), suggesting a possible hijacking of the host mitochondrial machinery and mitochondrial dysfunction in the infected host neurons, which could predispose the neurons to neurodegenerative diseases like Alzheimer’s disease, as seen in Table S3.

From analyses of the downregulated pathways, biological processes, and molecular functions altered in the infected neurons, metabolism was the major depleted component in the infected neurons (Figure 4B,C). Although we are not the first group to show hypometabolism following SARS-CoV-2 infection [32,98,99,100], which can be associated with cognitive decline [98], to our knowledge, this is the first study reporting neuronal metabolism, particularly lipid metabolism, being dysregulated after SARS-CoV-2 infection. A 2021 study performed proteomic and metabolomic profiling of COVID-19 patient plasma complemented with cell culture data and showed that the host metabolism pathways are hijacked by the SARS-CoV-2 virus [101]. The authors also showed that fatty acid metabolism was downregulated in the human lung epithelial Calu-3 cells after SARS-CoV-2 infection, which supports our observation. Another serum proteomic study in COVID-19 patients, both disease and recovery stage, showed disturbances in cholesterol metabolism [30], once again supporting our observation of disrupted cholesterol metabolism (Figure 4B). ELOVL1 (fatty acid elongase) has been associated with viral replication, as decreased ELOVL1 indicates disrupted viral replication [102]. The lack of replications of SARS-CoV-2 in the neurons is perhaps due to the decreased expression of ELOVL1 in infected neurons (Figure 2C) as a compensatory mechanism to evade the virus. Furthermore, ELOVL1 deficiency can lead to neurological defects such as hypomyelination [103] that would render the neuronal firing slow and less efficient and, therefore, could explain the fatigue experienced by COVID-19 patients, particularly in long COVID. Our proteomic profiling of infected neurons also revealed downregulation of HMGCS1 (Hydroxy-Methylglutaryl-CoA Synthase), which is involved in cholesterol biosynthesis and was noted to be downregulated in multiple cell lines infected with SARS-CoV-2 [104]. In fact, as a regulator of cholesterol synthesis/metabolism, HMGCS1 shows reduced expression in Alzheimer’s disease [105]. Therefore, the alteration of these metabolism-linked proteins in infected neurons indicates that the virus may disrupt neuronal metabolism, thereby predisposing the neurons to future neurological pathologies. 

We acknowledge that there are a few limitations to our study. Although our iPSC-derived neuronal model has unique advantages in studying brain-related pathologies and associated diseases, in the human brain, other support cells, such as the astrocytes and the microglia, contribute to the health of the neurons. Therefore, our study reveals neuronal pathologies after SARS-CoV-2 infection, but our findings do not cover the significance of the interplay between these different types of brain cells and their impact on regulating neuronal health in the presence of SARS-CoV-2. With studies showing astrocytes are more vulnerable than neurons, including both astrocytes and neurons in the proteomic analysis, would have revealed more significant pathological pathways induced after COVID-19. Due to limited time and resources, we could not pursue the astrocyte infection or astrocyte–neuron co-culture and the following proteomic analyses post-infection. Further phenotypic analyses of the neurons with the pre-conditioned media from either Calu-3 cells or astrocytes perhaps would have revealed lysosomal and mitochondrial changes in more detail. Finally, the pre-conditioned media from Calu-3 cells or astrocytes/neurons could have been used for a cytokine array or lipidomic/metabolomic analysis. Despite the existing limitations, our study displays unique neuroproteomic changes after SARS-CoV-2 infection that may answer some questions raised from the neurological symptoms in patients suffering from long COVID.

## 5. Conclusions

Although there is some overlap between the findings from our study and already published NeuroCOVID studies involving other cell types or body fluids, a limited number of studies have focussed on proteomic changes in neurons after exposure to SARS-CoV-2. Here, we report some unique protein changes in the neuroproteome post-SARS-CoV-2 infection. We confirmed that the limited viral infection was sufficient to drive the neurons toward apoptosis; however, the most striking finding was the disrupted lipid metabolism in the infected neurons. Long-term detrimental effects on the human brain due to lipid metabolism disruption are evident from the numerous studies in neurodegenerative diseases like Alzheimer’s disease and ageing (reviewed in [106]). Furthermore, altered mitochondrial function in the infected neurons, as suggested by our study, could also predispose neurons to neurodegeneration. Therefore, the death of the neurons post-SARS-CoV-2 infection could be due to a combined effect of mitochondrial dysfunction and disruption of metabolism. To summarise, our study displays unique neuroproteomic changes after SARS-CoV-2 infection that may answer some questions raised from the neurological symptoms in patients suffering from long COVID. 

## Data Availability

All Supplementary Materials include all the data generated in this study leading to the manuscript.

## Supplementary Materials

The following supporting information can be downloaded at: https://www.mdpi.com/article/10.3390/biom13111597/s1. Figure S1: Expression of possible SARS-CoV-2 entry points in the iPSC-derived i^3^Ns—*ACE2*, *ASGR1*, *BSG*, *NRP1*, *TMEM106* and *TMPRSS2.* Figure S2: (**A**) iPSCs were differentiated into astrocytes (i^3^As) and infected on Day 17 followed by immunocytochemistry analysis on Day 18. (**B**) iAs showed limited infection 24 h post-infection, as shown by the white arrow and the inset. Figure S3: Strategy for mass spectrometric analysis of infected neurons. (1) iPSCs were differentiated into i^3^Ns that were infected (2) either directly with the virus or treated with pre-conditioned media from infected Calu-3 cells. 24 h post-infection, the i^3^Ns were lysed and used for mass spectrometric analysis (3). Created with BioRender.com Figure S4: Mass spectrometric analysis identified neuroproteomic changes. A. Upregulated and downregulated proteins with a fold change of > 2 and *p*-value < 0.05 in i^3^Ns directly infected with SARS-CoV-2 compared to uninfected i^3^Ns. (**B**) Upregulated and downregulated proteins with a fold change of > 2 and *p*-value < 0.05 in i^3^Ns treated with pre-conditioned media from SARS-CoV-2 infected Calu-3 cells compared to i^3^Ns treated with media from uninfected Calu-3 cells. Figure S5: Analysis of upregulated proteins in infected i^3^Ns. (**A**) Protein–protein interaction of all upregulated proteins in infected i^3^Ns with extended interactors from the 1st shell showed more interactions compared to Figure 3B. (**B**) Highlighted proteins are involved in apoptotic pathways in i^3^Ns infected with SARS-CoV-2. Figure S6: Analysis of downregulated proteins in infected i^3^Ns. (**A**) Protein–protein interaction of all downregulated proteins in infected i^3^Ns with extended interactors from the 1st shell showed more interactions compared to Figure 4A. (**B**–**E**) Highlighted proteins are involved in metabolism (**B**), axon guidance (**C**), cholesterol biosynthesis (**D**), and call response to stress (**E**) in i^3^Ns infected with SARS-CoV-2. Table S1: Differentially expressed protein list in i^3^Ns infected with SARS-CoV-2. Table S2: Differentially expressed protein list in i^3^Ns treated with pre-conditioned media from SARS-CoV-2-infected Calu-3 cells. Table S3: Upregulated pathways in infected i^3^Ns (direct infection + pre-conditioned media). Table S4: Upregulated biological processes in infected i^3^Ns (direct infection + pre-conditioned media). Table S5: Upregulated molecular functions in infected i^3^Ns (direct infection + pre-conditioned media). Table S6: Downregulated pathways in infected i^3^Ns (direct infection + pre-conditioned media). Table S7: Downregulated biological processes in infected i^3^Ns (direct infection + pre-conditioned media). Table S8: Downregulated molecular functions in infected i^3^Ns (direct infection + pre-conditioned media).
