# Supplementary material for: Neuroproteomic Analysis after SARS-CoV-2 Infection Reveals Overrepresented Neurodegeneration Pathways and Disrupted Metabolic Pathways
Source: Biomolecules. 2023 Oct 30;13(11):1597. doi: 10.3390/biom13111597 (PMC10669333; doi:10.3390/biom13111597)
Supplement: Supplementary file 1 [file biomolecules-13-01597-s001.zip › biomolecules-2648729-supplementary.pdf]

Figure S1

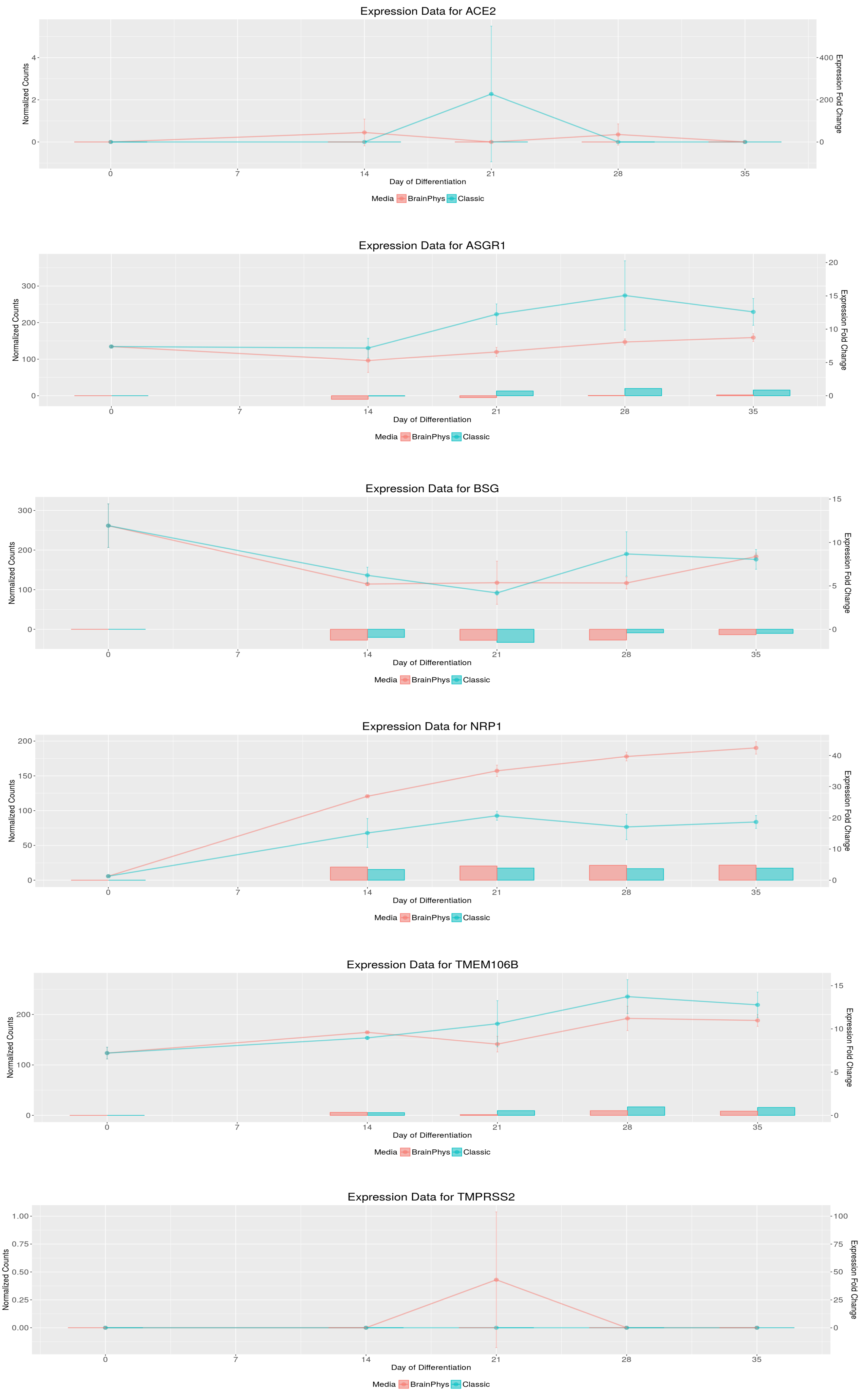
Expression Data for ACE2
Normalized Counts
Expression Fold Change
Day of Differentiation
0
7
14
21
28
35
Media
BrainPhys
Classic
Expression Data for ASGR1
Normalized Counts
Expression Fold Change
Day of Differentiation
Media
BrainPhys
Classic
Expression Data for BSG
Normalized Counts
Expression Fold Change
Day of Differentiation
Media
BrainPhys
Classic
Expression Data for NRP1
Normalized Counts
Expression Fold Change
Day of Differentiation
Media
BrainPhys
Classic
Expression Data for TMEM106B
Normalized Counts
Expression Fold Change
Day of Differentiation
Media
BrainPhys
Classic
Expression Data for TMPRSS2
Normalized Counts
Expression Fold Change
Day of Differentiation
Media
BrainPhys
Classic

Figure S2

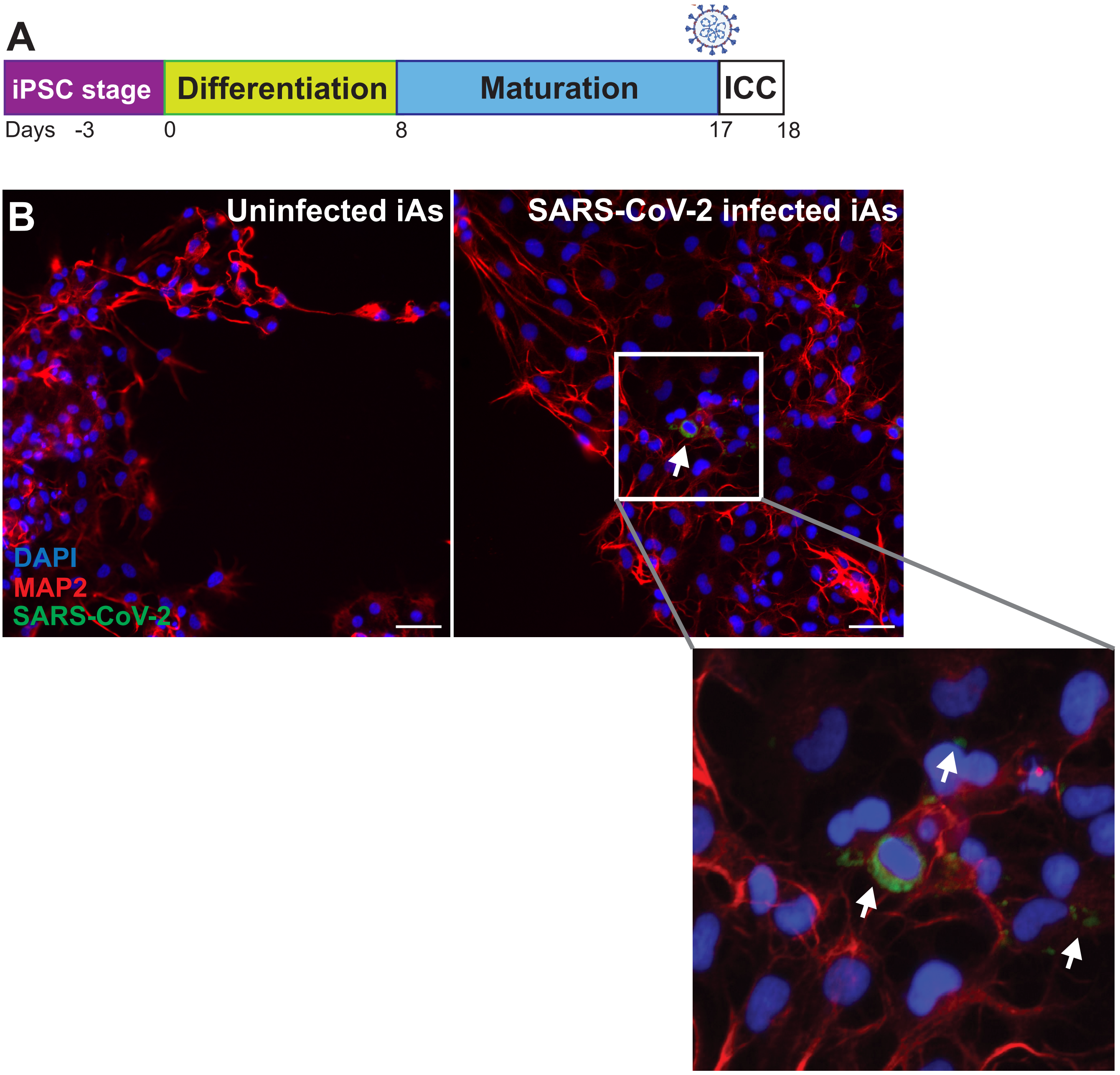
A
iPSC stage
Differentiation
Maturation
ICC
Days -3
0
8
17
18
B
Uninfected iAs
SARS-CoV-2 infected iAs
DAPI
MAP2
SARS-CoV-2

Figure S3

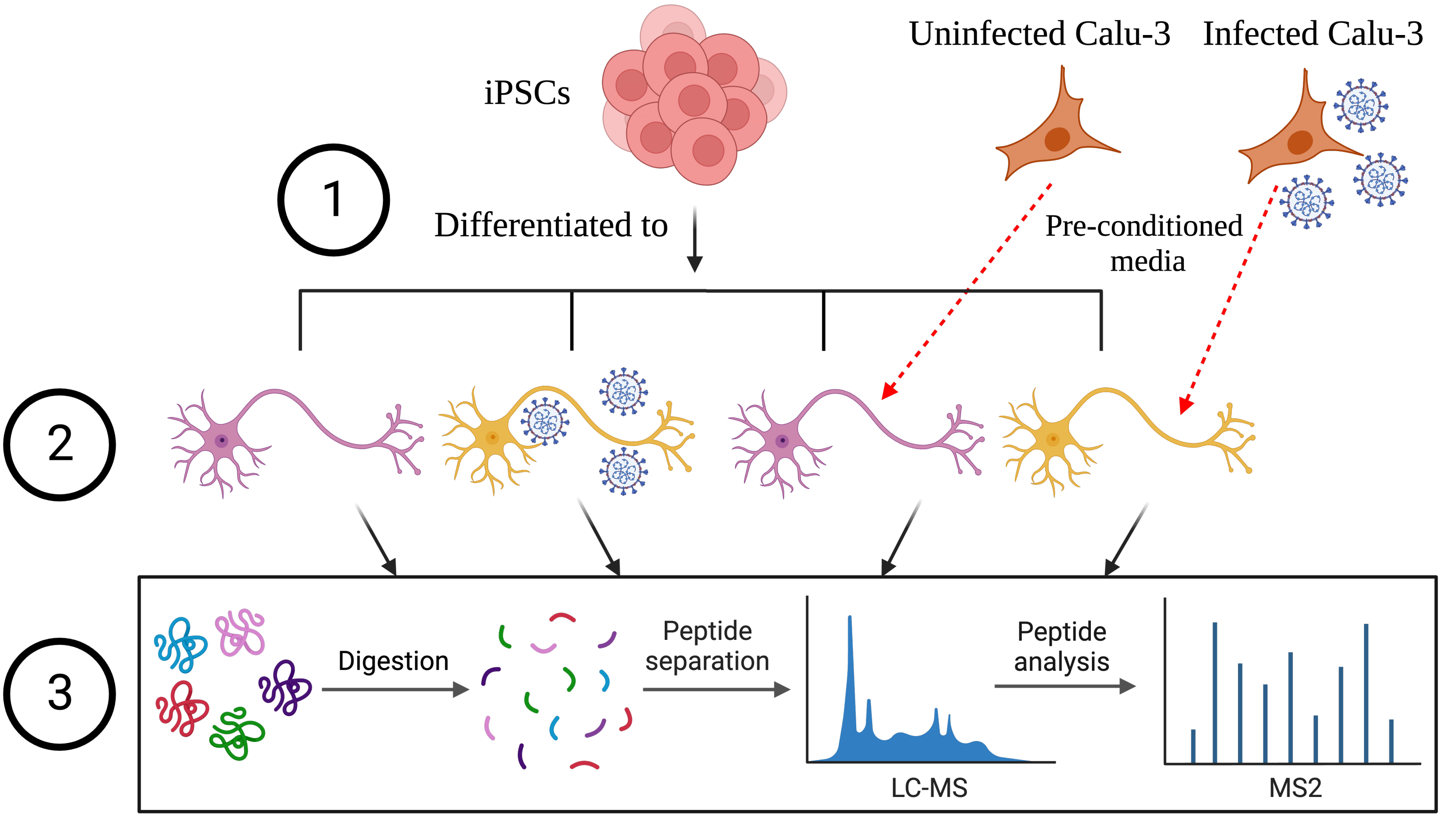

iPSCs
Uninfected Calu-3
Infected Calu-3
1
Differentiated to
Pre-conditioned media
2
3
Digestion
Peptide separation
Peptide analysis
LC-MS
MS2

Figure S4

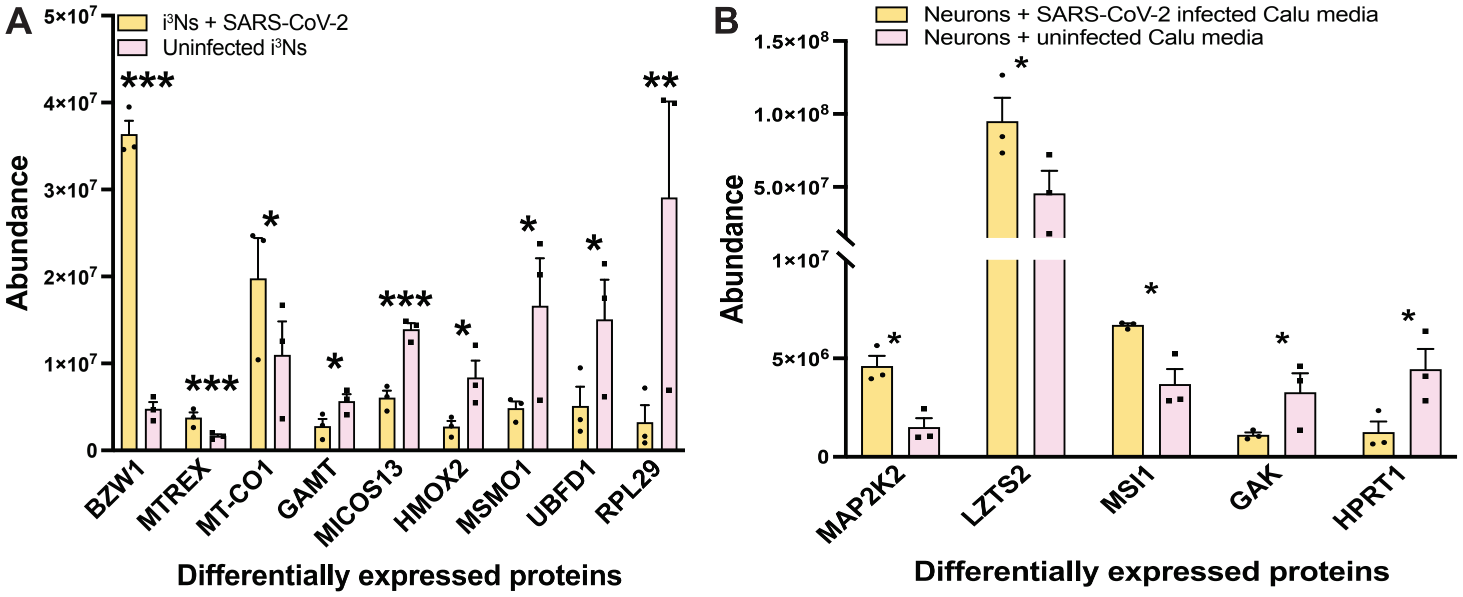

A
i³Ns + SARS-CoV-2
Uninfected i³Ns
Abundance
5×10^7
4×10^7
3×10^7
2×10^7
1×10^7
0
BZW1
MTREX
MT-CO1
GAMT
MICOS13
HMOX2
MSMO1
UBFD1
RPL29
Differentially expressed proteins
B
Neurons + SARS-CoV-2 infected Calu media
Neurons + uninfected Calu media
Abundance
1.5×10^8
1.0×10^8
5.0×10^7
1×10^7
5×10^6
0
MAP2K2
LZTS2
MSI1
GAK
HPRT1
Differentially expressed proteins

Figure S5

A

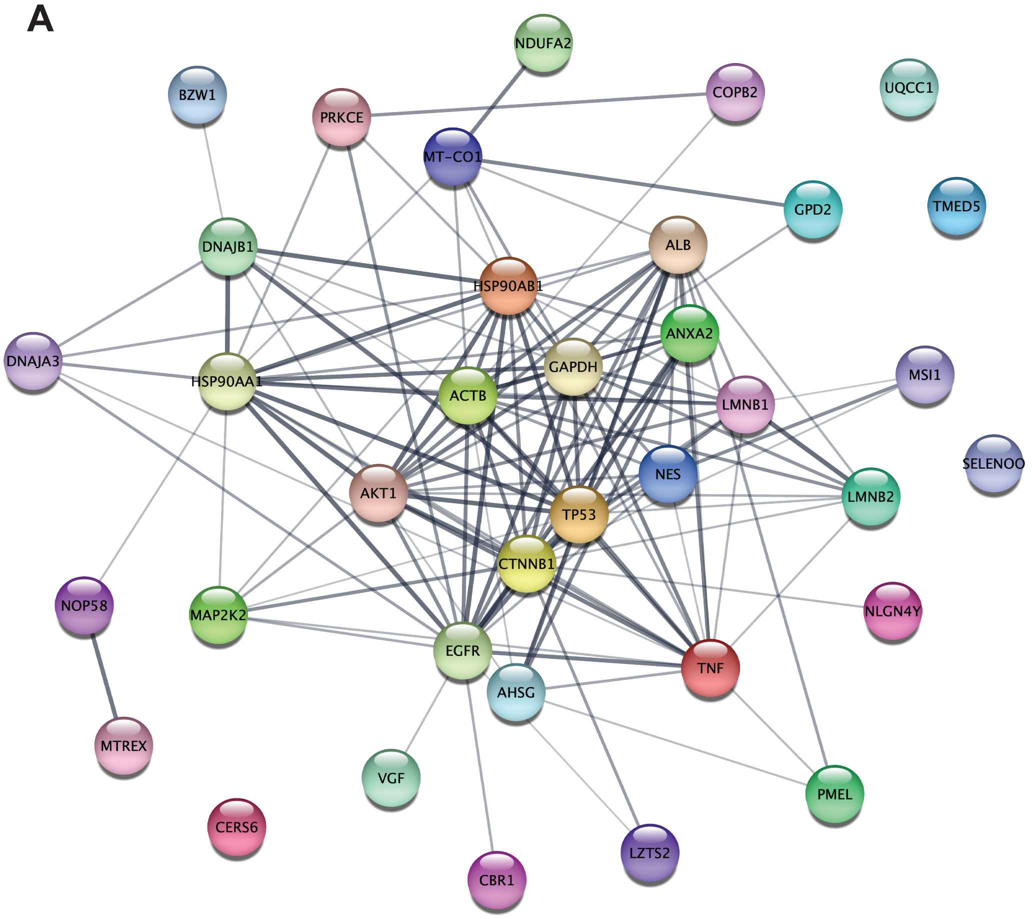
NDUFA2
BZW1
COPB2
UQCC1
PRKCE
MT-CO1
GPD2
TMED5
DNAJB1
ALB
HSP90AB1
ANXA2
DNAJA3
GAPDH
MSI1
HSP90AA1
ACTB
LMNB1
SELENOO
NES
AKT1
LMNB2
TP53
CTNNB1
NOP58
NLGN4Y
MAP2K2
EGFR
TNF
AHSG
MTREX
VGF
PMEL
CERS6
LZTS2
CBR1

B

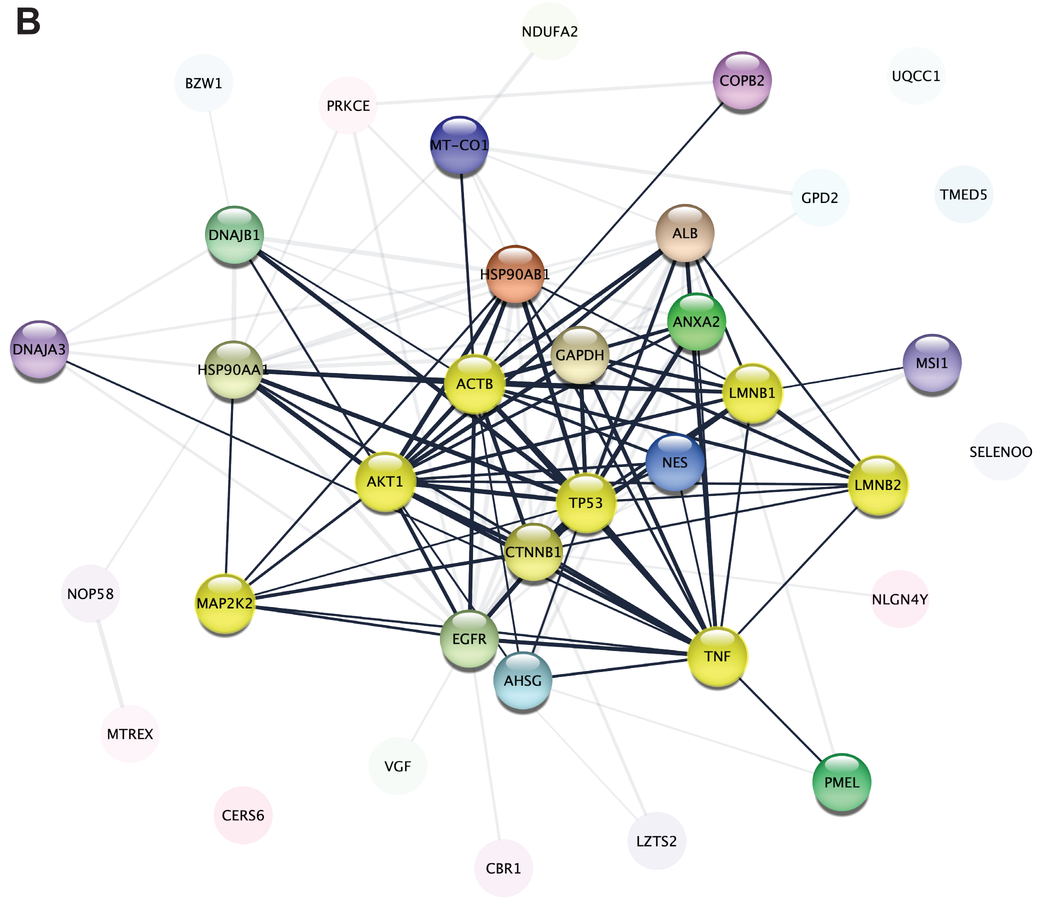
NDUFA2
BZW1
COPB2
UQCC1
PRKCE
MT-CO1
GPD2
TMED5
DNAJB1
ALB
HSP90AB1
ANXA2
DNAJA3
GAPDH
MSI1
HSP90AA1
ACTB
LMNB1
SELENOO
NES
AKT1
LMNB2
TP53
CTNNB1
NOP58
NLGN4Y
MAP2K2
EGFR
TNF
AHSG
MTREX
VGF
PMEL
CERS6
LZTS2
CBR1

Figure S6

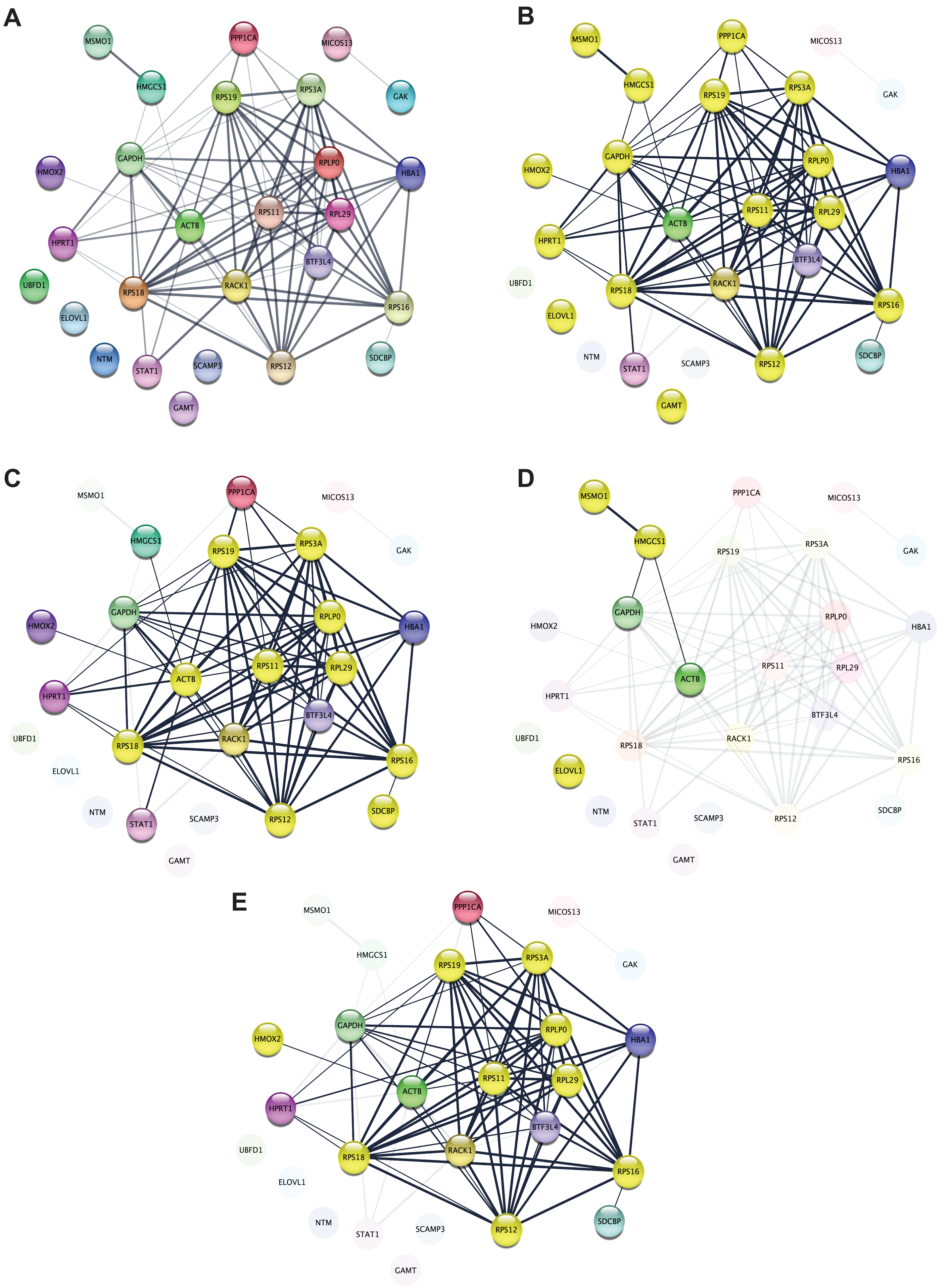
A
MSMO1
PPP1CA
MICOS13
HMGCS1
RPS19
RPS3A
GAK
GAPDH
RPLP0
HMOX2
HBA1
RPS11
RPL29
ACTB
HPRT1
BTF3L4
UBFD1
RPS18
RACK1
RPS16
ELOVL1
NTM
STAT1
SCAMP3
RPS12
SDCBP
GAMT
B
C
D
E

Table S1

Gene Symbol	Accession	Description	Coverage [%]	# Peptides	# AAs	MW [kDa]	Abundances (Neurons infected 10MOI,Exp_ Rep_1	Abundances (Neurons infected 10MOI,Exp_ Rep_2	Abundances (Neurons infected 10MOI,Exp_ Rep_3	Abundances (Neurons uninfected, Exp_Rep_1	Abundances (Neurons uninfected, Exp_Rep_2	Abundances (Neurons uninfected, Exp_Rep_3	P-VALUE (Neurons infected 10MOI) / (Neurons uninfected)	Fold change (Neurons infected 10MOI) / (Neurons uninfected)	Log2 Fold change (Neurons infected 10MOI) / (Neurons uninfected)
BZW1	Q7L1Q6	Basic leucine zipper and W2 domain-containing protein 1 OS=Homo sapiens OX=9606 GN=BZW1 PE=1 SV=1	9	2	419	48	39486128.3	34616019.3	34919740.4	6201765.6	4672663.43	3410889.53	**0.000**	**8.004**	2.973
MTREX	P42285	Exosome RNA helicase MTR4 OS=Homo sapiens OX=9606 GN=MTREX PE=1 SV=3	2	1	1042	117.7	4789450.76	2637481.57	3817221.27	2032091.45	1271455.72	1628985.98	**0.000**	**2.258**	1.173
MT-CO1	P00395	Cytochrome c oxidase subunit 1 OS=Homo sapiens OX=9606 GN=MT-CO1 PE=1 SV=1	6	1	513	57	24709217.6	10420581	24133118.9	16716230.4	3621365.45	12558610.7	**0.023**	**2.093**	1.007
VGF	O15240	Neurosecretory protein VGF OS=Homo sapiens OX=9606 GN=VGF PE=1 SV=2	6	2	615	67.2	7761896.05	7967594.75	9547882.34	4643209.68	3879001.67	4251967.79	**0.002**	**1.991**	0.983
NDUFA2	O43678	NADH dehydrogenase [ubiquinone] 1 alpha subcomplex subunit 2 OS=Homo sapiens OX=9606 GN=NDUFA2 PE=1 SV=3	21	1	99	10.9	2672175.63	2117426.52	5029526.18	1019275.27	1192451.96	3332394.72	**0.017**	**1.969**	0.937
ANXA2	P07355	Annexin A2 OS=Homo sapiens OX=9606 GN=ANXA2 PE=1 SV=2	44	12	339	38.6	34276527.5	30773273.7	38066348	17168255.5	18478491.1	19360306.4	**0.000**	**1.876**	0.907
PRKCE	Q02156	Protein kinase C epsilon type OS=Homo sapiens OX=9606 GN=PRKCE PE=1 SV=1	3	1	737	83.6	210828543	310165728	291681870	139481579	178434549	127263210	**0.008**	**1.847**	0.867
GPD2	P43304	Glycerol-3-phosphate dehydrogenase, mitochondrial OS=Homo sapiens OX=9606 GN=GPD2 PE=1 SV=3	8	4	727	80.8	11413484.1	12591676.2	13149524.1	7869757.48	5511900.84	9462593.51	**0.033**	**1.708**	0.733
NLGN4Y	B4DHI3	Neuroligin-4, Y-linked OS=Homo sapiens OX=9606 GN=NLGN4Y PE=1 SV=1	3	2	836	94.1	17819041.2	16585098.5	20763161.2	10074374.5	8235224.78	16576860.7	**0.025**	**1.679**	0.717
UQCC1	Q9NVA1	Ubiquinol-cytochrome-c reductase complex assembly factor 1 OS=Homo sapiens OX=9606 GN=UQCC1 PE=1 SV=3	3	1	299	34.6	3620803.88	6643312.84	4697289.26	2842521.23	3520293.11	2526488.8	**0.018**	**1.673**	0.720
NES	P48681	Nestin OS=Homo sapiens OX=9606 GN=NES PE=1 SV=2	2	2	1621	177.3	15177266.6	17211570.7	13043688.3	8992645.74	11069650.8	8018230.73	**0.000**	**1.623**	0.700
CBR1	P16152	Carbonyl reductase [NADPH] 1 OS=Homo sapiens OX=9606 GN=CBR1 PE=1 SV=3	5	1	277	30.4	14453136.2	8911700.71	13041726.2	9058721.79	6342068.59	7939479.53	**0.001**	**1.548**	0.627
TMED5	Q9Y3A6	Transmembrane emp24 domain-containing protein 5 OS=Homo sapiens OX=9606 GN=TMED5 PE=1 SV=1	13	2	229	26	52730652.6	43547293.2	61160430.4	28972625.5	34355348.2	40921677.9	**0.017**	**1.528**	0.593
STAT1	A0A669KB52	Signal transducer and activator of transcription OS=Homo sapiens OX=9606 GN=STAT1 PE=1 SV=1	3	1	636	74.2	1934368.47	4101113.5	4472186.42	4390522.16	5330106.64	7203409.25	**0.032**	**0.610**	-0.750
ELOVL1	Q9BW60	Elongation of very long chain fatty acids protein 1 OS=Homo sapiens OX=9606 GN=ELOVL1 PE=1 SV=1	5	1	279	32.6	680999.535	959414.616	644299.681	1371777.95	1499629.6	1056888.77	**0.002**	**0.582**	-0.787
NTM	F8W8Y1	Neurotrimin OS=Homo sapiens OX=9606 GN=NTM PE=1 SV=1	22	2	179	19.7	3234971.77	4522111.14	7371502.5	6049996.93	10005356.2	11273020	**0.005**	**0.547**	-0.887
HBA1	P69905	Hemoglobin subunit alpha OS=Homo sapiens OX=9606 GN=HBA1 PE=1 SV=2	28	3	142	15.2	144015582	149574201	176545366	234202829	325328369	389003680	**0.002**	**0.510**	-0.987
GAMT	A0A1W2PR3	Guanidinoacetate N-methyltransferase OS=Homo sapiens OX=9606 GN=GAMT PE=1 SV=1	7	1	213	23.8	1247650.41	2886592.4	4177745.19	4131471.03	5866569.04	6942449.91	**0.017**	**0.465**	-1.160
MICOS13	A0A140TA86	MICOS complex subunit MIC13 OS=Homo sapiens OX=9606 GN=MICOS13 PE=1 SV=1	37	2	140	15.4	4530397.16	7385723.86	6197023.83	12451770	14867775.4	14385952.6	**0.001**	**0.431**	-1.230
HMOX2	I3L4P8	Heme oxygenase 2 (Fragment) OS=Homo sapiens OX=9606 GN=HMOX2 PE=1 SV=8	12	1	130	15.1	3823451.9	2795420.41	1530055.18	5459667.15	12106288.4	7476229.88	**0.044**	**0.379**	-1.637
MSMO1	Q15800	Methylsterol monooxygenase 1 OS=Homo sapiens OX=9606 GN=MSMO1 PE=1 SV=1	5	1	293	35.2	5407125.7	5834836.15	3241866.24	20216718	23768156	5790270.14	**0.013**	**0.357**	-1.590
UBFD1	O14562	Ubiquitin domain-containing protein UBFD1 OS=Homo sapiens OX=9606 GN=UBFD1 PE=1 SV=2	4	1	309	33.4	3548875.91	2215916.93	9500912.01	21448638.8	6163848.17	17509686.5	**0.030**	**0.356**	-1.653
RPL29	A0A3B3ITT5	60S ribosomal protein L29 OS=Homo sapiens OX=9606 GN=RPL29 PE=1 SV=1	9	1	167	18.5	7178780.24	863801.931	1630011.9	40283875.7	6945582.96	39958680.5	**0.006**	**0.114**	-3.373

Table S2

Gene Symbol	Accession	Description	Coverage [%]	# Peptides	# AAs	MW [kDa]	Abundances (Neurons with infected Calu media,Exp_Rep_1	Abundances (Neurons with infected Calu media,Exp_Rep_2	Abundances (Neurons with infected Calu media,Exp_Rep_3	Abundances (Neurons with uninfected Calu media,Exp_Rep_1	Abundances (Neurons with uninfected Calu media,Exp_Rep_2	Abundances (Neurons with uninfected Calu media,Exp_Rep_3	P-VALUE: (Neurons with infected Calu media) / (Neurons with uninfected Calu media)	Fold change (Neurons with infected Calu media) / (Neurons with uninfected Calu media)	Log2 fold change (Neurons with infected Calu media) / (Neurons with uninfected Calu media)
MAP2K2	P36507	Dual specificity mitogen-activated protein kinase kinase 2 OS=Homo sapiens OX=9606 GN=MAP2K2 PE=1 SV=1	5	1	400	44.4	5653888.67	4159245.55	3970646.21	1042435.62	1003634.73	2434397.73	**0.030**	**3.733**	1.733
LZTS2	Q9BRK4	Leucine zipper putative tumor suppressor 2 OS=Homo sapiens OX=9606 GN=LZTS2 PE=1 SV=2	2	1	669	72.7	84517369.1	73340460	126646442	46195665	17830094.2	72053187.1	**0.036**	**2.567**	1.240
MSI1	O43347	RNA-binding protein Musashi homolog 1 OS=Homo sapiens OX=9606 GN=MSI1 PE=1 SV=1	9	1	362	39.1	6804254.22	6760166.56	6476667.35	5243876.05	2846748.3	2934859.56	**0.028**	**1.960**	0.923
DNAJA3	Q96EY1	DnaJ homolog subfamily A member 3, mitochondrial OS=Homo sapiens OX=9606 GN=DNAJA3 PE=1 SV=2	5	1	480	52.5	6462586.9	5646233.87	2311836.1	2785893.25	2631984.59	1653034.78	**0.015**	**1.955**	0.930
SELENOO	Q9BVL4	Protein adenylyltransferase SelO, mitochondrial OS=Homo sapiens OX=9606 GN=SELENOO PE=1 SV=3	6	2	669	73.4	2876831.92	5579663.14	3970370.77	1602161.92	3748370.02	1861917.56	**0.005**	**1.806**	0.833
CERS6	Q6ZMG9	Ceramide synthase 6 OS=Homo sapiens OX=9606 GN=CERS6 PE=1 SV=1	3	1	384	44.9	1388753.52	2674939.61	1714841.32	1079409.37	1094769.84	1026007.63	**0.042**	**1.800**	0.797
NOP58	Q9Y2X3	Nucleolar protein 58 OS=Homo sapiens OX=9606 GN=NOP58 PE=1 SV=1	16	6	529	59.5	8522768.54	15802475.4	8839014.1	3745806.47	10225516.4	6879113.32	**0.041**	**1.702**	0.727
VGF	O15240	Neurosecretory protein VGF OS=Homo sapiens OX=9606 GN=VGF PE=1 SV=2	6	2	615	67.2	5461131.39	8201110.46	6655932.54	3288199.73	6215759.67	3186210.25	**0.019**	**1.690**	0.730
COPB2	H0Y938	Coatomer subunit beta' OS=Homo sapiens OX=9606 GN=COPB2 PE=1 SV=2	5	2	898	101.5	13372858.1	11447768.9	12473261.2	9689714.9	6522807.11	7661504.21	**0.003**	**1.588**	0.657
DNAJB1	M0R080	DnaJ homolog subfamily B member 1 (Fragment) OS=Homo sapiens OX=9606 GN=DNAJB1 PE=1 SV=1	10	1	183	20.5	12334044.1	14624292	11305948.3	7223190.42	9347241.25	7776859.5	**0.001**	**1.576**	0.653
LMNB1	P20700	Lamin-B1 OS=Homo sapiens OX=9606 GN=LMNB1 PE=1 SV=2	41	19	586	66.4	84645294.1	90119621.9	81956084.5	48411104.8	51891289.9	66826146	**0.021**	**1.570**	0.633
AHSG	P02765	Alpha-2-HS-glycoprotein OS=Homo sapiens OX=9606 GN=AHSG PE=1 SV=2	7	4	367	39.3	6415775.49	6524458.67	10641872	4550059.88	5310860.46	5296049.54	**0.046**	**1.549**	0.603
LMNB2	Q03252	Lamin-B2 OS=Homo sapiens OX=9606 GN=LMNB2 PE=1 SV=4	17	9	620	69.9	35138942.9	33051307.3	33791618	17661152.9	24990270.3	25961846.2	**0.042**	**1.538**	0.590
PMEL	F8W1D1	Melanocyte protein PMEL (Fragment) OS=Homo sapiens OX=9606 GN=PMEL PE=1 SV=1	7	1	299	32.3	12761519.1	11846212.7	7162404.16	9120020.87	6392801.43	5712367.16	**0.028**	**1.502**	0.567
SCAMP3	O14828	Secretory carrier-associated membrane protein 3 OS=Homo sapiens OX=9606 GN=SCAMP3 PE=1 SV=3	14	3	347	38.3	13719353.4	9866450.06	10406195.5	16669116.1	17604646.3	17491095.8	**0.023**	**0.659**	-0.623
PPP1CA	P62136	Serine/threonine-protein phosphatase PP1-alpha catalytic subunit OS=Homo sapiens OX=9606 GN=PPP1CA PE=1 SV=1	41	9	330	37.5	11116841.8	11819417.5	9173960.17	18666180.7	16408816.3	15277357	**0.002**	**0.639**	-0.653
BTF3L4	Q96K17	Transcription factor BTF3 homolog 4 OS=Homo sapiens OX=9606 GN=BTF3L4 PE=1 SV=1	36	4	158	17.3	16903959.7	16751032	18495287.3	28894466	23514097.6	31721928.4	**0.002**	**0.627**	-0.680
HMGCS1	Q01581	Hydroxymethylglutaryl-CoA synthase, cytoplasmic OS=Homo sapiens OX=9606 GN=HMGCS1 PE=1 SV=2	22	9	520	57.3	18809078.4	21354662.2	18944774.2	37129943.3	33238420.8	31048987.9	**0.002**	**0.586**	-0.777
SDCBP	O00560	Syntenin-1 OS=Homo sapiens OX=9606 GN=SDCBP PE=1 SV=1	21	3	298	32.4	10957080.5	9677270.18	8881010.4	24410310.5	19712278.3	22097586.7	**0.000**	**0.447**	-1.170
GAK	O14976	Cyclin-G-associated kinase OS=Homo sapiens OX=9606 GN=GAK PE=1 SV=2	2	1	1311	143.1	1366859.65	1036911.65	905710.775	3859034.64	4592503.73	1349427.39	**0.036**	**0.417**	-1.410
HPRT1	P00492	Hypoxanthine-guanine phosphoribosyltransferase OS=Homo sapiens OX=9606 GN=HPRT1 PE=1 SV=2	5	1	218	24.6	725887.506	2345069.58	654027.274	2836594.42	4087270.98	6388343.84	**0.048**	**0.311**	-2.020

Table S3

Category	GO	Upregulated pathways in infected i3Ns	LogP	Hits
KEGG Pathway	hsa05208	Chemical carcinogenesis - reactive oxygen species	-4.4	CBR1|MT-CO1|NDUFA2|MAP2K2
GO Biological Processes	GO:0022904	respiratory electron transport chain	-3.9	GPD2|MT-CO1|NDUFA2
KEGG Pathway	hsa04071	Sphingolipid signaling pathway	-3.8	PRKCE|MAP2K2|CERS6
KEGG Pathway	hsa04210	Apoptosis	-3.7	LMNB1|MAP2K2|LMNB2
GO Biological Processes	GO:0006091	generation of precursor metabolites and energy	-3.6	GPD2|MT-CO1|NDUFA2|VGF
GO Biological Processes	GO:0022900	electron transport chain	-3.4	GPD2|MT-CO1|NDUFA2
GO Biological Processes	GO:0061024	membrane organization	-3.4	ANXA2|LMNB1|DNAJA3|NLGN4Y|LMNB2
GO Biological Processes	GO:0009266	response to temperature stimulus	-3.4	DNAJB1|VGF|DNAJA3
GO Biological Processes	GO:1903034	regulation of response to wounding	-3.4	ANXA2|PRKCE|MAP2K2
GO Biological Processes	GO:0045333	cellular respiration	-3.2	GPD2|MT-CO1|NDUFA2
GO Biological Processes	GO:0015980	energy derivation by oxidation of organic compounds	-2.8	GPD2|MT-CO1|NDUFA2
KEGG Pathway	hsa05010	Alzheimer disease	-2.4	MT-CO1|NDUFA2|MAP2K2
GO Biological Processes	GO:0007005	mitochondrion organization	-2.2	NDUFA2|DNAJA3|UQCC1
GO Biological Processes	GO:0099536	synaptic signaling	-2.2	PRKCE|VGF|NLGN4Y
KEGG Pathway	hsa05022	Pathways of neurodegeneration - multiple diseases	-2.1	MT-CO1|NDUFA2|MAP2K2
GO Biological Processes	GO:0050804	modulation of chemical synaptic transmission	-2.1	PRKCE|VGF|NLGN4Y
GO Biological Processes	GO:0099177	regulation of trans-synaptic signaling	-2.1	PRKCE|VGF|NLGN4Y
GO Biological Processes	GO:0010638	positive regulation of organelle organization	-2	ANXA2|PRKCE|NES
Reactome Gene Sets	R-HSA-9006934	Signaling by Receptor Tyrosine Kinases	-2	PRKCE|MAP2K2|VGF

Table S4

**Upregulated Biological Processes in infected i3Ns**	p-value	q-value
Protein Localization To Nuclear Envelope (GO:0090435)	5.82E-05	0.01277448
Nuclear Migration (GO:0007097)	0.000192831	0.01277448
Nuclear Envelope Organization (GO:0006998)	0.000218367	0.01277448
Postsynaptic Membrane Organization (GO:0001941)	0.000218367	0.01277448
Heterochromatin Formation (GO:0031507)	0.000786914	0.03682757
Membrane Assembly (GO:0071709)	0.001109993	0.03775719
Heterochromatin Organization (GO:0070828)	0.001169059	0.03775719
Membrane Organization (GO:0061024)	0.001290844	0.03775719
Nucleus Organization (GO:0006997)	0.001486539	0.03865002
Negative Regulation Of Gene Expression, Epigenetic (GO:0045814)	0.001992369	0.04662145
Positive Regulation Of Cellular Metabolic Process (GO:0031325)	0.002396892	0.05098843
Positive Regulation Of Cytoskeleton Organization (GO:0051495)	0.004708244	0.07917454
Mitochondrial Respiratory Chain Complex Assembly (GO:0033108)	0.006066898	0.07917454
rRNA Metabolic Process (GO:0016072)	0.006198298	0.07917454
Positive Regulation Of Vesicle Fusion (GO:0031340)	0.006483689	0.07917454
Regulation Of Plasma Membrane Repair (GO:1905684)	0.006483689	0.07917454
snoRNA Localization (GO:0048254)	0.006483689	0.07917454
ncRNA Processing (GO:0034470)	0.007438995	0.07917454
rRNA Processing (GO:0006364)	0.007583244	0.07917454
Peptidyl-Serine Autophosphorylation (GO:0036289)	0.009065844	0.07917454
Postsynaptic Membrane Assembly (GO:0097104)	0.009065844	0.07917454
Positive Regulation Of Fibroblast Migration (GO:0010763)	0.009065844	0.07917454
Endoplasmic Reticulum To Golgi Vesicle-Mediated Transport (GO:0006888)	0.009734268	0.07917454
Golgi Ribbon Formation (GO:0090161)	0.010354502	0.07917454

Regulation Of Vesicle Fusion (GO:0031338)	0.010354502	0.07917454
Vitamin K Metabolic Process (GO:0042373)	0.010354502	0.07917454
Cyclooxygenase Pathway (GO:0019371)	0.010354502	0.07917454
Presynaptic Membrane Assembly (GO:0097105)	0.010354502	0.07917454
Protein Localization To Nucleus (GO:0034504)	0.010900111	0.07917454
Quinone Metabolic Process (GO:1901661)	0.011641548	0.07917454
Negative Regulation Of Sodium Ion Transmembrane Transport (GO:1902306)	0.011641548	0.07917454
Postsynapse Assembly (GO:0099068)	0.011641548	0.07917454
Presynaptic Membrane Organization (GO:0097090)	0.011641548	0.07917454
Negative Regulation Of Inclusion Body Assembly (GO:0090084)	0.012926986	0.07917454
Regulation Of Inclusion Body Assembly (GO:0090083)	0.012926986	0.07917454
Positive Regulation Of Protein Depolymerization (GO:1901881)	0.012926986	0.07917454
Negative Regulation Of Sodium Ion Transmembrane Transporter Activity (GO:2000650)	0.012926986	0.07917454
Regulation Of Cytoplasmic Transport (GO:1903649)	0.014210816	0.07917454
Regulation Of Stress-Activated Protein Kinase Signaling Cascade (GO:0070302)	0.014210816	0.07917454
Mitochondrial Respiratory Chain Complex III Assembly (GO:0034551)	0.014210816	0.07917454
Respiratory Chain Complex III Assembly (GO:0017062)	0.014210816	0.07917454
Presynapse Organization (GO:0099172)	0.014210816	0.07917454
Negative Regulation Of Receptor Internalization (GO:0002091)	0.016773662	0.08987931
Ribosome Biogenesis (GO:0042254)	0.01716994	0.08987931
Peptidyl-Serine Phosphorylation (GO:0018105)	0.017800851	0.08987931
Membrane Raft Organization (GO:0031579)	0.018052682	0.08987931
Neuron Cell-Cell Adhesion (GO:0007158)	0.018052682	0.08987931
Regulation Of Cellular Response To Heat (GO:1900034)	0.019330102	0.0923111
Presynapse Assembly (GO:0099054)	0.019330102	0.0923111
Maturation Of 5.8S rRNA (GO:0000460)	0.020605924	0.09454483
Regulation Of Golgi Organization (GO:1903358)	0.020605924	0.09454483

Prostaglandin Biosynthetic Process (GO:0001516)	0.021880151	0.09660293
Toll-Like Receptor 4 Signaling Pathway (GO:0034142)	0.021880151	0.09660293
Response To Organophosphorus (GO:0046683)	0.023152783	0.09995444
Regulation Of Early Endosome To Late Endosome Transport (GO:2000641)	0.024423824	0.09995444
Receptor Clustering (GO:0043113)	0.024423824	0.09995444
Lipopolysaccharide-Mediated Signaling Pathway (GO:0031663)	0.025693274	0.09995444
Positive Regulation Of Protein Processing (GO:0010954)	0.025693274	0.09995444
Regulation Of Fibroblast Migration (GO:0010762)	0.025693274	0.09995444
Fc-gamma Receptor Signaling Pathway Involved In Phagocytosis (GO:0038096)	0.025693274	0.09995444
Endomembrane System Organization (GO:0010256)	0.026614404	0.09995444
Golgi Vesicle Transport (GO:0048193)	0.026866135	0.09995444
snRNA Metabolic Process (GO:0016073)	0.026961136	0.09995444
Regulation Of Translation (GO:0006417)	0.0281394	0.09995444
Fc Receptor Mediated Stimulatory Signaling Pathway (GO:0002431)	0.028227412	0.09995444
Regulation Of Neural Precursor Cell Proliferation (GO:2000177)	0.028227412	0.09995444
Response To Purine-Containing Compound (GO:0014074)	0.030755212	0.09995444
Fc-gamma Receptor Signaling Pathway (GO:0038094)	0.030755212	0.09995444
Neuromuscular Junction Development (GO:0007528)	0.030755212	0.09995444
ERK1 And ERK2 Cascade (GO:0070371)	0.030755212	0.09995444
Fat-Soluble Vitamin Metabolic Process (GO:0006775)	0.030755212	0.09995444
Negative Regulation Of Ion Transmembrane Transporter Activity (GO:0032413)	0.030755212	0.09995444
Camera-Type Eye Development (GO:0043010)	0.033276689	0.10360518
Negative Regulation Of Receptor-Mediated Endocytosis (GO:0048261)	0.033276689	0.10360518
Negative Regulation Of Catalytic Activity (GO:0043086)	0.033276689	0.10360518
Chaperone Cofactor-Dependent Protein Refolding (GO:0051085)	0.034535061	0.10360518
Myeloid Cell Development (GO:0061515)	0.034535061	0.10360518
Positive Regulation Of Response To Wounding (GO:1903036)	0.034535061	0.10360518
Positive Regulation Of Neural Precursor Cell Proliferation (GO:2000179)	0.037047083	0.1097344

Presynaptic Endocytosis (GO:0140238)	0.03955282	0.115692
'De Novo' Post-Translational Protein Folding (GO:0051084)	0.040803336	0.11646119
intra-Golgi Vesicle-Mediated Transport (GO:0006891)	0.042052286	0.11646119
ncRNA Catabolic Process (GO:0034661)	0.042052286	0.11646119
Positive Regulation Of Small Molecule Metabolic Process (GO:0062013)	0.042052286	0.11646119
Positive Regulation Of Organelle Organization (GO:0010638)	0.043299673	0.11646119
Positive Regulation Of Carbohydrate Metabolic Process (GO:0045913)	0.043299673	0.11646119
Mitochondrial Electron Transport, NADH To Ubiquinone (GO:0006120)	0.043299673	0.11646119
Regulation Of Receptor Internalization (GO:0002090)	0.045789763	0.11697245
Regulation Of Trans-Synaptic Signaling (GO:0099177)	0.045789763	0.11697245
Melanosome Organization (GO:0032438)	0.04703247	0.11697245
Regulation Of Sodium Ion Transmembrane Transporter Activity (GO:2000649)	0.04703247	0.11697245
Positive Regulation Of Signaling (GO:0023056)	0.04703247	0.11697245
Regulation Of Ossification (GO:0030278)	0.04703247	0.11697245
G2/M Transition Of Mitotic Cell Cycle (GO:0000086)	0.048273621	0.11697245
Response To cAMP (GO:0051591)	0.048273621	0.11697245
Positive Regulation Of ATP-dependent Activity (GO:0032781)	0.049513218	0.11697245
Positive Regulation Of Cytokinesis (GO:0032467)	0.049513218	0.11697245

Table S5

**Upregulated Molecular Functions in infected i3Ns**	**p-value**	**q-value**
ATPase Binding (GO:0051117)	0.00403523	0.09085148
Sphingosine N-acyltransferase Activity (GO:0050291)	0.00777557	0.09085148
Cadherin Binding (GO:0045296)	0.00796435	0.09085148
Endopeptidase Inhibitor Activity (GO:0004866)	0.00925288	0.09085148
Phospholipase Inhibitor Activity (GO:0004859)	0.01164155	0.09085148
MAP-kinase Scaffold Activity (GO:0005078)	0.01292699	0.09085148
Neurexin Family Protein Binding (GO:0042043)	0.01421082	0.09085148
MAP Kinase Kinase Activity (GO:0004708)	0.01805268	0.09085148
Protein Kinase C Activity (GO:0004697)	0.01805268	0.09085148
Oxidoreductase Activity, Acting On NAD(P)H, Quinone Or Similar Compound As Acceptor (GO:0016655)	0.0193301	0.09085148
Cadherin Binding Involved In Cell-Cell Adhesion (GO:0098641)	0.02188015	0.09348792
Actin Monomer Binding (GO:0003785)	0.0294921	0.10426696
Adenylyltransferase Activity (GO:0070566)	0.03201674	0.10426696
RNA Binding (GO:0003723)	0.03293571	0.10426696
Protein Serine/Threonine/Tyrosine Kinase Activity (GO:0004712)	0.03327669	0.10426696
snoRNA Binding (GO:0030515)	0.03955282	0.10526315
NADH Dehydrogenase (Ubiquinone) Activity (GO:0008137)	0.04205229	0.10526315
NADH Dehydrogenase (Quinone) Activity (GO:0050136)	0.04329967	0.10526315
Hsp70 Protein Binding (GO:0030544)	0.04329967	0.10526315
N-acyltransferase Activity (GO:0016410)	0.04703247	0.10526315
Peptidase Inhibitor Activity (GO:0030414)	0.04703247	0.10526315

Table S6

Category	GO	Downregulated pathways in infected i3Ns	LogP	Hits
GO Biological Processes	GO:0044283	small molecule biosynthetic process	-5.4	GAMT|HMGCS1|HPRT1|MSMO1|ELOVL1
Reactome Gene Sets	R-HSA-556833	Metabolism of lipids	-3.2	HMGCS1|PPP1CA|MSMO1|ELOVL1
GO Biological Processes	GO:0009150	purine ribonucleotide metabolic process	-3.1	HMGCS1|HPRT1|ELOVL1
GO Biological Processes	GO:0009259	ribonucleotide metabolic process	-3	HMGCS1|HPRT1|ELOVL1
GO Biological Processes	GO:0019693	ribose phosphate metabolic process	-3	HMGCS1|HPRT1|ELOVL1
GO Biological Processes	GO:0006979	response to oxidative stress	-2.9	HBA1|HMOX2|STAT1
GO Biological Processes	GO:0006163	purine nucleotide metabolic process	-2.8	HMGCS1|HPRT1|ELOVL1
WikiPathways	WP3888	VEGFA-VEGFR2 signaling	-2.8	PPP1CA|SDCBP|STAT1
GO Biological Processes	GO:0072521	purine-containing compound metabolic process	-2.7	HMGCS1|HPRT1|ELOVL1
GO Biological Processes	GO:1901615	organic hydroxy compound metabolic process	-2.7	HMGCS1|HPRT1|MSMO1
GO Biological Processes	GO:0009117	nucleotide metabolic process	-2.6	HMGCS1|HPRT1|ELOVL1
GO Biological Processes	GO:0006753	nucleoside phosphate metabolic process	-2.6	HMGCS1|HPRT1|ELOVL1
GO Biological Processes	GO:0032787	monocarboxylic acid metabolic process	-2.6	GAMT|MSMO1|ELOVL1
GO Biological Processes	GO:0010035	response to inorganic substance	-2.5	HBA1|PPP1CA|STAT1
GO Biological Processes	GO:0090407	organophosphate biosynthetic process	-2.5	HMGCS1|HPRT1|ELOVL1
GO Biological Processes	GO:0055086	nucleobase-containing small molecule metabolic process	-2.5	HMGCS1|HPRT1|ELOVL1
GO Biological Processes	GO:0008610	lipid biosynthetic process	-2.4	HMGCS1|MSMO1|ELOVL1
Reactome Gene Sets	R-HSA-2262752	Cellular responses to stress	-2	HBA1|HMOX2|RPL29
GO Biological Processes	GO:0043603	amide metabolic process	-2	HMGCS1|RPL29|ELOVL1

Table S7

Downregulated Biological Processes in infected i3Ns	p-value	q-value
Secondary Alcohol Biosynthetic Process (GO:1902653)	0.00018563	0.01928305
Cholesterol Biosynthetic Process (GO:0006695)	0.00020167	0.01928305
Sterol Biosynthetic Process (GO:0016126)	0.00025372	0.01928305
Cholesterol Metabolic Process (GO:0008203)	0.00145547	0.0589797
Monocarboxylic Acid Metabolic Process (GO:0032787)	0.00301943	0.0589797
Synaptic Vesicle Uncoating (GO:0016191)	0.00424314	0.0589797
Mesenchymal Cell Differentiation Involved In Kidney Development (GO:0072161)	0.00424314	0.0589797
Regulation Of Extracellular Exosome Assembly (GO:1903551)	0.00424314	0.0589797
Interleukin-27-Mediated Signaling Pathway (GO:0070106)	0.00424314	0.0589797
Interleukin-9-Mediated Signaling Pathway (GO:0038113)	0.00508974	0.0589797
Response To Lead Ion (GO:0010288)	0.00508974	0.0589797
Clathrin Coat Disassembly (GO:0072318)	0.00508974	0.0589797
GMP Biosynthetic Process (GO:0006177)	0.00508974	0.0589797
Substrate-Dependent Cell Migration, Cell Extension (GO:0006930)	0.00508974	0.0589797
Macrophage Derived Foam Cell Differentiation (GO:0010742)	0.00508974	0.0589797
Cellular Response To Interleukin-9 (GO:0071355)	0.00508974	0.0589797
Regulation Of Dopamine Metabolic Process (GO:0042053)	0.00593566	0.0589797
Foam Cell Differentiation (GO:0090077)	0.00593566	0.0589797
IMP Biosynthetic Process (GO:0006188)	0.00593566	0.0589797
Purine Ribonucleoside Monophosphate Catabolic Process (GO:0009169)	0.00593566	0.0589797
GMP Metabolic Process (GO:0046037)	0.00678091	0.0589797
Purine Nucleobase Metabolic Process (GO:0006144)	0.00762547	0.0589797
Type II Interferon-Mediated Signaling Pathway (GO:0060333)	0.00762547	0.0589797
Positive Regulation Of Mesenchymal Cell Proliferation (GO:0002053)	0.00762547	0.0589797
Alpha-Linolenic Acid Metabolic Process (GO:0036109)	0.00762547	0.0589797
Peptidyl-Serine Dephosphorylation (GO:0070262)	0.00762547	0.0589797

Cell Differentiation Involved In Metanephros Development (GO:0072202)	0.00762547	0.0589797
IMP Metabolic Process (GO:0046040)	0.00762547	0.0589797
Nitrogen Compound Transport (GO:0071705)	0.00828509	0.0589797
Oxygen Transport (GO:0015671)	0.00846937	0.0589797
Metanephric Mesenchyme Development (GO:0072075)	0.00846937	0.0589797
Heme Catabolic Process (GO:0042167)	0.00846937	0.0589797
Carbon Dioxide Transport (GO:0015670)	0.00931258	0.0589797
Golgi To Lysosome Transport (GO:0090160)	0.00931258	0.0589797
Purine Ribonucleoside Monophosphate Metabolic Process (GO:0009167)	0.00931258	0.0589797
Regulation Of Mesenchymal Cell Proliferation (GO:0010464)	0.00931258	0.0589797
Fatty Acid Elongation (GO:0030497)	0.01015513	0.05936843
Porphyrin-Containing Compound Catabolic Process (GO:0006787)	0.01015513	0.05936843
Cristae Formation (GO:0042407)	0.01015513	0.05936843
Clathrin Coat Assembly (GO:0048268)	0.01099699	0.05969797
Negative Regulation Of Receptor Internalization (GO:0002091)	0.01099699	0.05969797
Substrate-Dependent Cell Migration (GO:0006929)	0.01099699	0.05969797
Positive Regulation Of Exosomal Secretion (GO:1903543)	0.01183819	0.06276993
Gas Transport (GO:0015669)	0.01267871	0.06569877
acetyl-CoA Metabolic Process (GO:0006084)	0.01351856	0.06680742
Peptidyl-Threonine Dephosphorylation (GO:0035970)	0.01435773	0.06680742
Golgi To Vacuole Transport (GO:0006896)	0.01435773	0.06680742
Purine-Containing Compound Biosynthetic Process (GO:0072522)	0.01435773	0.06680742
Long-Chain fatty-acyl-CoA Biosynthetic Process (GO:0035338)	0.01435773	0.06680742
Regulation Of Exosomal Secretion (GO:1903541)	0.01519624	0.06793613
Response To Organophosphorus (GO:0046683)	0.01519624	0.06793613
Linoleic Acid Metabolic Process (GO:0043651)	0.01603407	0.06897676
Nucleotide Biosynthetic Process (GO:0009165)	0.01603407	0.06897676
Hydrogen Peroxide Catabolic Process (GO:0042744)	0.01687123	0.0712341

Positive Regulation Of Interferon-Alpha Production (GO:0032727)	0.01770773	0.07209575
Purine Nucleotide Biosynthetic Process (GO:0006164)	0.01770773	0.07209575
Cellular Response To Interferon-Beta (GO:0035458)	0.01854355	0.07315248
Long-Chain fatty-acyl-CoA Metabolic Process (GO:0035336)	0.0193787	0.07315248
Entrainment Of Circadian Clock By Photoperiod (GO:0043153)	0.0193787	0.07315248
Photoperiodism (GO:0009648)	0.02021319	0.07315248
Response To Purine-Containing Compound (GO:0014074)	0.02021319	0.07315248
Positive Regulation Of Cellular Response To Transforming Growth Factor Beta Stimulus (GO:1903846)	0.02021319	0.07315248
Positive Regulation Of Transforming Growth Factor Beta Receptor Signaling Pathway (GO:0030511)	0.02021319	0.07315248
fatty-acyl-CoA Biosynthetic Process (GO:0046949)	0.021047	0.07336286
Purine Ribonucleotide Catabolic Process (GO:0009154)	0.021047	0.07336286
Regulation Of Interferon-Alpha Production (GO:0032647)	0.02188015	0.07336286
Negative Regulation Of Receptor-Mediated Endocytosis (GO:0048261)	0.02188015	0.07336286
Positive Regulation Of Erythrocyte Differentiation (GO:0045648)	0.02188015	0.07336286
Chaperone Cofactor-Dependent Protein Refolding (GO:0051085)	0.02271263	0.07397829
Clathrin-Dependent Endocytosis (GO:0072583)	0.02271263	0.07397829
Positive Regulation Of Defense Response To Virus By Host (GO:0002230)	0.02354445	0.07455741
Protein Localization To Golgi Apparatus (GO:0034067)	0.02354445	0.07455741
Response To Interferon-Beta (GO:0035456)	0.02437559	0.07613199
Positive Regulation Of Phosphate Metabolic Process (GO:0045937)	0.02520608	0.07638245
Receptor Signaling Pathway Via STAT (GO:0097696)	0.02603589	0.07638245
acyl-CoA Metabolic Process (GO:0006637)	0.02603589	0.07638245
'De Novo' Post-Translational Protein Folding (GO:0051084)	0.02686504	0.07638245
Mitochondrial Membrane Organization (GO:0007006)	0.02686504	0.07638245
Negative Regulation Of Proteasomal Ubiquitin-Dependent Protein Catabolic Process (GO:0032435)	0.02686504	0.07638245
Very Long-Chain Fatty Acid Metabolic Process (GO:0000038)	0.02686504	0.07638245
Purine Nucleotide Metabolic Process (GO:0006163)	0.02852135	0.07638245
Inner Mitochondrial Membrane Organization (GO:0007007)	0.02852135	0.07638245

One-Carbon Compound Transport (GO:0019755)	0.02934851	0.07638245
Receptor Signaling Pathway Via JAK-STAT (GO:0007259)	0.03017501	0.07638245
Positive Regulation Of Myeloid Cell Differentiation (GO:0045639)	0.03017501	0.07638245
Regulation Of Erythrocyte Differentiation (GO:0045646)	0.03017501	0.07638245
Regulation Of Receptor Internalization (GO:0002090)	0.03017501	0.07638245
Negative Regulation Of Endothelial Cell Proliferation (GO:0001937)	0.03100085	0.07638245
Regulation Of Defense Response To Virus By Host (GO:0050691)	0.03100085	0.07638245
Type I Interferon-Mediated Signaling Pathway (GO:0060337)	0.03100085	0.07638245
Positive Regulation Of Exocytosis (GO:0045921)	0.03100085	0.07638245
Tumor Necrosis Factor-Mediated Signaling Pathway (GO:0033209)	0.03182602	0.07638245
Cellular Response To Type I Interferon (GO:0071357)	0.03182602	0.07638245
Response To cAMP (GO:0051591)	0.03182602	0.07638245
Response To Peptide (GO:1901652)	0.03182602	0.07638245
Negative Regulation Of Proteasomal Protein Catabolic Process (GO:1901799)	0.03265053	0.07674558
Negative Regulation Of Ubiquitin-Dependent Protein Catabolic Process (GO:2000059)	0.03265053	0.07674558
Synaptic Vesicle Endocytosis (GO:0048488)	0.03347438	0.07709252
Endothelial Cell Migration (GO:0043542)	0.03347438	0.07709252
Negative Regulation Of I-kappaB kinase/NF-kappaB Signaling (GO:0043124)	0.0351201	0.07928103
Organic Hydroxy Compound Biosynthetic Process (GO:1901617)	0.0351201	0.07928103
Regulation Of Cellular Component Size (GO:0032535)	0.03594198	0.08034089
Epithelial Cell Migration (GO:0010631)	0.03840364	0.08409095
Response To Hydrogen Peroxide (GO:0042542)	0.03840364	0.08409095
Positive Regulation Of Smooth Muscle Cell Proliferation (GO:0048661)	0.03922288	0.08409095
Positive Regulation Of Epithelial To Mesenchymal Transition (GO:0010718)	0.03922288	0.08409095
Unsaturated Fatty Acid Metabolic Process (GO:0033559)	0.04085939	0.08409095
Positive Regulation Of Pathway-Restricted SMAD Protein Phosphorylation (GO:0010862)	0.04085939	0.08409095
Interferon-Mediated Signaling Pathway (GO:0140888)	0.04085939	0.08409095
Steroid Biosynthetic Process (GO:0006694)	0.04167665	0.08409095

Positive Regulation Of Cell Death (GO:0010942)	0.04167665	0.08409095
Negative Regulation Of Cell Projection Organization (GO:0031345)	0.04167665	0.08409095
Blood Circulation (GO:0008015)	0.04167665	0.08409095
Protein Homotetramerization (GO:0051289)	0.04412453	0.08757379
Regulation Of Smooth Muscle Cell Proliferation (GO:0048660)	0.04493918	0.08757379
Negative Regulation Of Neuron Projection Development (GO:0010977)	0.04493918	0.08757379
Endoplasmic Reticulum Organization (GO:0007029)	0.04493918	0.08757379
Response To Peptide Hormone (GO:0043434)	0.04575318	0.08840445
post-Golgi Vesicle-Mediated Transport (GO:0006892)	0.04656653	0.0892199
Membrane Lipid Biosynthetic Process (GO:0046467)	0.04737922	0.09002051
Renal System Development (GO:0072001)	0.04819126	0.09080667
Regulation Of Cell Death (GO:0010941)	0.04900265	0.09157872
Sterol Metabolic Process (GO:0016125)	0.04981338	0.09160438

Table S8

Downregulated Molecular Functions in infected i3Ns	p-value	q-value
Cadherin Binding (GO:0045296)	0.00012828	0.00538758
Heme Binding (GO:0020037)	0.00243818	0.05120184
Oxidoreductase Activity, Acting On Paired Donors, With Incorporation Or Reduction Of Molecular Oxygen, Another Compound As One Donor, And Incorporation Of One Atom Of Oxygen (GO:0016716)	0.00424314	0.05940402
Tumor Necrosis Factor Receptor Binding (GO:0005164)	0.00762547	0.07108588
Fatty Acid Synthase Activity (GO:0004312)	0.00846937	0.07108588
RNA Polymerase II Core Promoter Sequence-Specific DNA Binding (GO:0000979)	0.01015513	0.07108588
Histone Acetyltransferase Binding (GO:0035035)	0.01435773	0.07537811
Cadherin Binding Involved In Cell-Cell Adhesion (GO:0098641)	0.01435773	0.07537811
Oxidoreductase Activity, Acting On Paired Donors, With Incorporation Or Reduction Of Molecular Oxygen, Reduced Flavin Or Flavoprotein As One Donor, And Incorporation Of One Atom Of Oxygen (GO:0016712)	0.021047	0.08386461
Tumor Necrosis Factor Receptor Superfamily Binding (GO:0032813)	0.02271263	0.08386461
Ubiquitin-Like Protein Ligase Binding (GO:0044389)	0.02452902	0.08386461
Frizzled Binding (GO:0005109)	0.02769353	0.08386461
Transcription Coactivator Binding (GO:0001223)	0.02852135	0.08386461
Core Promoter Sequence-Specific DNA Binding (GO:0001046)	0.02934851	0.08386461
Methyltransferase Activity (GO:0008168)	0.03100085	0.08386461
Promoter-Specific Chromatin Binding (GO:1990841)	0.0351201	0.08386461
Transcription Corepressor Binding (GO:0001222)	0.0351201	0.08386461
Cell-Cell Adhesion Mediator Activity (GO:0098632)	0.03594198	0.08386461
S-adenosylmethionine-dependent Methyltransferase Activity (GO:0008757)	0.04167665	0.09094938
Phosphoric Ester Hydrolase Activity (GO:0042578)	0.04330923	0.09094938
Protein Serine/Threonine Phosphatase Activity (GO:0004722)	0.04575318	0.09150636
